# Post-stroke lesion correlates of errors in verbal and spatial production tasks

**DOI:** 10.3389/fpsyg.2025.1517876

**Published:** 2025-04-28

**Authors:** Antonino Visalli, Natasha Maldonado, Mete Dadak, Heinrich Lanfermann, Karin Weißenborn, Bruno Kopp

**Affiliations:** ^1^Department of Neurology, Hannover Medical School, Hannover, Germany; ^2^Department of Biomedical, Metabolic and Neuroscience, University of Modena and Reggio Emilia, Reggio Emilia, Italy; ^3^Institute of Diagnostic and Interventional Neuroradiology, Hannover Medical School, Hannover, Germany

**Keywords:** design fluency, word-fragment completion, error monitoring, brain lateralization, stroke, voxel-based lesion-behavior mapping

## Abstract

**Introduction:**

Traditional lateralization models assign post-stroke verbal impairments to the left hemisphere and spatial impairments to the right hemisphere. When considering error measures, this dichotomy may be too simplistic, as performance monitoring may involve domain-general and domain-specific components. Furthermore, the error-monitoring hypothesis predicts domain-incongruent specialization, with left hemisphere dominance for spatial and right hemisphere dominance for verbal errors.

**Methods:**

We performed voxel-based lesion-behavior mapping in *N* = 110 acute stroke patients who completed a cognitively demanding, error-prone, five-point spatial design fluency task and a verbal word-fragment completion task.

**Results:**

Significant associations were found between lesion location and error rates in both tasks, spatial fluency (correlation = 0.36, *p* < 0.001) and verbal completion (correlation = 0.31, *p* = 0.001). Right inferior frontal lesions correlated with errors in both tasks. In addition, left frontal white matter (WM) lesions were associated with spatial errors, whereas right frontal WM lesions were associated with verbal errors. After adjusting for demographics, the left WM cluster remained significant for spatial errors and the right WM cluster for verbal errors, while the right inferior frontal association with spatial errors was no longer significant.

**Discussion:**

Post-stroke performance monitoring involves two distinct neural systems. One is a domain-general system, probably centered in the right inferior frontal region, that supports overall accuracy. The other is a widely distributed, reverse lateralized system, with left lesions associated with spatial accuracy and right lesions associated with verbal accuracy. This suggests that performance monitoring relies on more complex hemispheric interactions than traditional models suggest.

## 1 Introduction

This study investigated the neural basis of performance monitoring in patients with first-ever ischemic stroke. Performance monitoring involves the continuous evaluation of intended actions with actual performance, ensuring that behavior is adapted to the demands of the task (Ullsperger et al., 2014). Within this system, error monitoring detects errors and initiates post-error processing adjustments, including increased cognitive control, to prevent errors from occurring in the future. Thus, deficiencies in these processes can compromise the accuracy of performance. We examined error rates in modified spatial (five-point design) fluency (Regard et al., 1982; Lee et al., 1997) and verbal (word-fragment) completion (Tulving et al., 1982; Lustig and Hasher, 2001a, 2001b) tasks that do not provide external feedback. Using voxel-based lesion-behavior mapping (Bates et al., 2003; Rorden and Karnath, 2004; Vaidya et al., 2019; Pustina and Mirman, 2022), we analyzed lesion correlates of productivity and accuracy, using the former as a proxy for processing efficiency and the latter as a proxy for monitoring processes. Our general goal was to determine whether monitoring relies on domain-general or domain-specific mechanisms, as described below.

Working memory provides the task-relevant information needed to compare actions against intended outcomes (Logie et al., 2020). The dominant model (Baddeley, 2007) proposes two domain-specific storage subsystems - a left-lateralized phonological loop for verbal material and a right-lateralized visuospatial sketchpad for spatial content - that operate under the control of a domain-general central executive that allocates attention and cognitive resources (Fodor, 1983). In addition, Petrides (2000) distinguishes between storage and monitoring processes: whereas posterior brain regions support the maintenance of information in working memory, the mid-dorsolateral prefrontal cortex (PFC) is critical for monitoring the contents of working memory, particularly during self-structured tasks that require continuous evaluation and updating. This distinction is critical to understanding why stroke patients with prefrontal lesions may exhibit impairments in performance monitoring, as disruptions in these monitoring processes can lead to difficulties in accurately tracking one’s own behavior.

Compared to functional neuroimaging, lesion-behavior studies in stroke patients provide unique insights into how focal brain damage relates to deficits in executive function (e.g., Tsuchida and Fellows, 2013; Krämer et al., 2013). In this study, we investigated the neural correlates of productivity and accuracy in stroke patients performing both spatial and verbal production tasks. While productivity may be affected by domain-specific lesions - left lateralized for verbal tasks and right lateralized for spatial tasks—competing models of neural mechanisms for performance monitoring propose different sources of error. One model (which is consistent with Petrides’ view; Petrides, 2000) predicts that damage to a domain-general central monitoring process will facilitate errors in both types of tasks. A second model, consistent with the phonological and visuospatial subsystems of Baddeley and colleagues’ model (Baddeley, 2007), proposes that errors arise from lesions within the two specialized domain-specific systems, left lateralized for verbal tasks and right lateralized for spatial tasks. A third model, referred to as the hemispheric monitoring (Zaidel, 1987) or error monitoring (Hochman and Eviatar, 2004, 2006) hypothesis, suggests that errors are related to damage in a specific error-monitoring system that exhibits the opposite, i.e., domain-incongruent, lateralization, left lateralized for spatial tasks and right lateralized for verbal tasks, as described below. The primary aim of this study was to distinguish between these three models by analyzing lesion-error correlations in both spatial and verbal tasks.

The neural organization of error monitoring remains controversial. It may be centrally organized, relying on a unified executive system; it may follow hemispheric independence (Zaidel et al., 1990), with each hemisphere monitoring its own errors; or it may adhere to the hemispheric monitoring hypothesis (Zaidel, 1987), where error monitoring emerges from interhemispheric comparison. The error-monitoring hypothesis argues that because error monitoring is cognitively demanding, it follows a domain-incongruent lateralization pattern (Hochman and Meiran, 2005). Indeed, in tasks dominated by one hemisphere, such as lexical decision (left hemisphere) and bar-graph judgment (right hemisphere), a reverse lateralization effect has been observed, with the non-dominant hemisphere detecting errors more efficiently (Hochman and Eviatar, 2004, 2006). This effect increases under high cognitive load, suggesting that the non-dominant hemisphere compensates for processing limitations of the dominant hemisphere (Banich, 1998). Furthermore, Hochman et al. (2011) demonstrated that intact interhemispheric communication is essential for effective error monitoring, as patients with partial callosal disconnection showed impairments.

Thus, there are three models of how performance monitoring occurs across the cerebral hemispheres. One model attributes monitoring to a central, domain-general executive system that monitors errors equally in both verbal and spatial tasks, predicting either no lateralization if organized bilaterally or possible right hemisphere dominance. A second model, based on hemispheric independence, suggests that each hemisphere monitors errors within its specialized domain, with the left hemisphere primarily monitoring verbal errors and the right hemisphere focusing on spatial errors. In contrast, hemispheric interaction hypotheses, including the error-monitoring hypothesis, propose a reverse lateralization effect under high cognitive load, in which the non-dominant hemisphere monitors the dominant one; specifically, the right hemisphere monitors verbal errors while the left monitors spatial errors.

The error-monitoring hypothesis has remained unexplored in stroke patients, who often exhibit cognitive impairments - with left hemisphere lesions affecting verbal performance and right hemisphere lesions affecting spatial function (Bradshaw and Nettleton, 1981; Miceli et al., 1981; Hellige, 2001; Moore and Demeyere, 2022; Vallesi, 2012; Rost et al., 2022). Existing research suggests that key regions such as the inferior PFC are critical for successful performance monitoring (Gehring and Knight, 2000; Ullsperger et al., 2002; Ullsperger and von Cramon, 2006). Although Ullsperger et al. (2002) focused on left-sided lesions and Ullsperger and von Cramon (2006) included patients with left- and right-sided lesions, the systematic investigation of lesion laterality remains an often neglected aspect in the neuropsychology of executive functions. In addition, Mandal et al. (2020) linked increased speech errors in post-stroke aphasia to lesions in left frontal white matter and dorsolateral PFC, while McCall et al. (2022) showed that disrupted connections between left posterior medial PFC and speech areas enhance speech errors. Finally, some studies suggest that right hemisphere lesions are also associated with higher error rates on verbal tasks (Iacoboni et al., 1997; Kaplan and Zaidel, 2001; Robinson et al., 2021).

This study investigates in stroke patients whether performance monitoring is associated with damage to domain-general central processes, domain-specific working memory subsystems, or a system dedicated to error monitoring that exhibits reverse (domain-incongruent) lateralization. To address this question, we analyzed lesion-error correlations in both spatial and verbal tasks, building on existing evidence that regions in the PFC and intact interhemispheric connectivity are key for effective performance monitoring. To test these hypotheses, we conducted a lesion-behavior study in stroke patients who had experienced their first ischemic event. Participants completed two production tasks: a spatial five-point design (5PD) fluency task and a verbal word-fragment completion (WFC) task. Using voxel-based lesion-behavior mapping, we examined how lesion location correlated with two performance measures - productivity (fluency) and accuracy (error rates) - across both task domains. This approach allowed us to investigate whether performance monitoring relies on domain-general, domain-specific, or domain-incongruent neural mechanisms.

Fluency tasks, which require individuals to generate as many unique responses as possible under time constraints, are popular in neuropsychological assessment of executive function. Verbal fluency (Thurstone and Thurstone, 1962) typically engages left hemisphere regions, including prefrontal and temporal cortices, which support language production and comprehension (Baldo et al., 2001; Henry and Crawford, 2004; Robinson et al., 2012; Cipolotti et al., 2020; Biesbroek et al., 2021; Godefroy et al., 2023). In contrast, design fluency (Jones-Gotman and Milner, 1977) has traditionally been associated with the right hemisphere due to its spatial nature, although evidence for this lateralization is less consistent (Baldo et al., 2001; Marin et al., 2017; Cipolotti et al., 2020). Most neuropsychological research on fluency emphasizes productivity, while accuracy (error rates) is less frequently examined due to its relative rarity. For example, Stuss et al. (1998) attempted to classify verbal fluency errors but found low overall error rates. Cipolotti et al. (2020) reported that damage to the left posterior medial PFC was associated with phonemic rule violations. Recent work by Robinson et al. (2021) showed that fluency errors - including repetitions and rule violations - are primarily associated with right lateral PFC lesions across a variety of fluency tasks (verbal, design, gesture, and ideational fluency).

To increase the sensitivity of design fluency to performance monitoring in stroke patients, we introduced a modified five-point design (m5PD) fluency task. This adaptation increased error susceptibility by lengthening task duration, introducing a movable window to increase working memory demands, strictly limiting the number of valid designs to enforce strategic problem solving, and withholding external error feedback. These modifications were designed to enhance the role of working memory and performance monitoring in design fluency performance in stroke patients.

Verbal completion tasks assess language processing. For example, word-fragment completion tasks require participants to add missing letters to word fragments (e.g., in our study, complete “s_t” with “set”). These tasks primarily engage a left-lateralized network: the visual word form area in the left ventral occipitotemporal cortex processes letter patterns, the left inferior parietal lobule supports phonological working memory, and the left inferior prefrontal and temporal cortices facilitate semantic retrieval. Sentence-stem completion tasks that require completing sentences with semantically incongruent words, such as Part B of the Hayling task (Burgess and Shallice, 1996), recruit additional right-hemisphere regions (Volle et al., 2012; Cipolotti et al., 2016). However, evidence for right-hemisphere involvement in nonverbal tasks such as picture completion remains inconclusive (Bilder, 2011; Chelune, 2017).

To increase the sensitivity of verbal completion to performance monitoring in stroke patients, we implemented a modified WFC (mWFC) paradigm with four key adjustments: extended task duration, a moving display window to make previous choices irreversible, ambiguous trigrams to increase the difficulty of finding unique final letters that transform the two consonants into valid nouns, and withholding of external error feedback. These modifications were designed to increase cognitive demands and performance monitoring in verbal completion performance in stroke patients.

This study investigates the neural correlates of cognitive productivity and accuracy in stroke patients using modified spatial and verbal production tasks. Although previous research has examined hemispheric specialization for spatial fluency and verbal completion, the relationship between productivity and accuracy in lesion studies remains poorly understood, in part due to the typically low error rates in standard tasks. Based on existing evidence, productivity in verbal completion may be associated with left hemisphere lesions, whereas productivity in spatial fluency may be associated with right hemisphere lesions, following traditional lateralization patterns. However, the neural basis of accuracy may go beyond conventional domain-congruent hemispheric specialization. Error rates in both tasks may be associated with damage to a central domain-general monitoring process, which may be either bilateral or right dominant. Alternatively, error rates may be associated with damage to domain-congruent working memory systems that follow typical lateralization patterns, i.e., verbal errors-left lesions, spatial errors-right lesions. Finally, error rates may be associated with damage to an error-monitoring system that shows a reverse lateralization effect, i.e., verbal errors-right lesions, spatial errors-left lesions. It is important to note that these mechanisms are not mutually exclusive and can coexist.

## 2 Materials and methods

### 2.1 Patient sample

Our patient sample consisted of 110 patients with acute ischemic stroke (73 men and 37 women) who were admitted to the Stroke Unit of the Department of Neurology at the Hannover Medical School. All patients in the study were native speakers of German language. The demographic information of the patient sample is shown in Table 1. The mean age was approximately 67 years, range 35 to 90 years, and the mean number of years of education was approximately 13 years, range 7 to 18 years. The lead physician of the Stroke Unit, KW, identified patients who appeared to be eligible for the study. Patients with medical records indicating a history of stroke or other neurological disease were not included. Patients with large cortical territory infarcts, including those involving the middle cerebral artery, were excluded from the study if clinically evident severe aphasic or neglect symptoms were present. All included patients had to have a first-ever ischemic stroke that occurred between 1 and 14 days before brain imaging and the behavioral assessment that was part of this study. 56 patients had a left hemisphere stroke, 51 patients had a right hemisphere stroke, and three patients had evidence of bilateral lesions. A single investigator, NM, performed the final neuropsychological screening for inclusion and the target neuropsychological assessments for all patients. This single-rater approach eliminates the inconsistent results that can occur when different raters are assessing the patients in a neuropsychological study. The screening included assessment of auditory sentence comprehension, visual word comprehension, and visual neglect, as described below.

**Table 1:** Summary of demographic and neuropsychological sample data.

	M	SD	Med	Min	Max
Age (in years)	67.4	11.2	68	35	90
Education (in years)	12.8	2.7	12	7	18
Handedness (EHI-SF; −100… + 100)	94.7	21.3	100	-50	100
Auditory sentence comprehension (BiAS-as; 0 … 6)	5.80	0.52	6	4	6
Visual word comprehension (BiAS-vw; 0 … 6)	5.94	0.31	6	4	6
Visual hemi-spatial neglect (LCT-CoC; −1 … + 1)	0.01	0.06	0	−0.04	0.64*
Depressive symptoms (BDI-FS; 0 … 21)	1.53	2.39	1	0	14

EHI SF, Edinburgh Handedness Inventory - Short Form (Veale, 2014); BiAS, Bielefeld Aphasie Screeening (Richter et al., 2006); LCT, Line Cancellation Task (modified from Albert, 1973), CoC, Center of Cancellation (Binder et al., 1992; Rorden and Karnath, 2010); BDI FSBeck Depression Inventory - Fast Screen (German version: Kliem and Brähler, 2013). *Only one patient showed some degree of hemi-spatial visual neglect (CoC = +0.64). All other CoC scores were in the range [−0.05, +0.07], with *n* = 100 CoC scores exactly equal to 0.

No formal sample size calculation was performed prior to the study. The sample size of 110 patients was based on the availability of eligible stroke patients at our institution during the maximum recruitment period of the study.

A separate control group was not included in this study. For lesion-symptom mapping analyses examining behavioral deficits following focal brain injury, it is not necessary to include a control group of neurologically intact individuals. The goal is to map associations between behavioral deficits and lesion locations within the patient sample itself using voxel-wise analysis techniques.

### 2.2 Neuropsychological screening

The Edinburgh Handedness Inventory - Short Form (EHI-SF) (Veale, 2014) simplifies the original Edinburgh Handedness Inventory (Oldfield, 1971) by assessing handedness with only four items, writing, throwing, toothbrush use, and spoon use. Responses can be “always right” (+100), “usually right” (+50), “both equal” (0), “usually left” (−50), or “always left” (−100). The EHI-SF laterality quotient is the average of these scores, resulting in a range from −100 (strongly left-handed) to +100 (strongly right-handed).

Auditory sentence comprehension was assessed using the auditory sentence subtest of the Bielefeld Aphasia Screening (BiAS-as) (Richter et al., 2006). This subtest consists of six items (SET B in the BiAS manual) in which the examiner reads simple sentences aloud (e.g., ‘DIE KATZE LIEGT NEBEN DEM STUHL’, ‘THE CAT LIES NEXT TO THE CHAIR’). The subject selects the target picture from three vertically arranged options: the target sentence, a near distractor, and a far distractor. Each item is scored as either correct (1) or incorrect (0), and the total BiAS-as score is the sum of the six items. A score of ≥ 4 was required for inclusion in the study to ensure sufficient auditory sentence comprehension to understand verbal task instructions.

The BiAS-vw subtest of the Bielefeld Aphasia Screening (Richter et al., 2006) was used to assess reading ability and visual word comprehension. This subtest consists of six items (SET E in the BiAS manual) in which the subject reads simple concrete target nouns (e.g., ‘TISCH’, table) and selects the target picture from three options: the target noun, a semantic distractor (e.g., ‘STUHL’, chair), and a phonemic distractor (e.g., ‘FISCH’, fish). Each item is scored as either correct (1) or incorrect (0), with the total BiAS-vw score being the sum of the six items. A score of ≥ 4 was required for inclusion in the study. This was to ensure sufficient reading and visual word comprehension skills for the verbal task of the study.

The Line Cancellation Task (LCT; modified from Albert, 1973) has been widely used to screen neurological patients for visual spatial hemi-neglect (Ferber and Karnath, 2001). The task consists of crossing out 41 short lines on a sheet of paper, arranged in six columns of six lines each, plus a central column of five lines. The Center of Cancellation (CoC) score assesses visual neglect by considering both the number and spatial distribution of line omissions (Binder et al., 1992; Rorden and Karnath, 2010). A CoC score of zero indicates no omissions or spatially unbiased omissions. CoC scores approach −1 for rightward neglect and +1 for leftward neglect.

The Beck Depression Inventory Fast Screen (BDI-FS; Beck et al., 2000; German version: BDI-FS-G; Kliem and Brähler, 2013) is an abbreviated version of the original Beck Depression Inventory, a 21-item questionnaire that assesses self-reported severity of depressive symptoms over the past 2 weeks. The BDI-FS includes seven items that focus on cognitive and affective symptoms: Sadness, Pessimism, Past Failures, Anhedonia, Self-Rejection, Self-Criticism, and Suicidal Thoughts. Each item is scored from 0 (lowest) to 3 (highest), with total scores ranging from 0 (no depressive symptoms) to 21 (severe depressive symptoms).

### 2.3 Generative tasks

#### 2.3.1 Modified five-point design fluency task

Spatial fluency tasks assess nonverbal fluency, visuospatial abilities, and executive function. One such task, the Five-Point Design (5PD) task (Regard et al., 1982), requires participants to generate as many unique designs as possible by connecting five fixed dots with straight lines while following specific rules. Performance is measured by the number of unique designs produced and errors such as rule violations or repetitions. The 5PD task is commonly used in neuropsychological assessments to evaluate frontal lobe function, particularly in individuals with executive dysfunction.

Our study introduced a modified version of the 5PD task (m5PD) with three major changes designed to increase cognitive demands.

1. Extended task duration (from 1–2 min to 5 min)

Longer duration increases the need for sustained attention and cognitive control, making executive deficits more apparent. It also provides a broader sample of performance, reducing the influence of momentary fluctuations in effort or strategy.

1. Movable viewing window (previous designs were hidden after completion)

This modification increases working memory demands and requires greater resistance to proactive interference-participants must track their progress without visual cues, relying instead on internal memory to avoid repetition. The examiner manually advances the window as each design is completed, ensuring controlled exposure to new items.

1. Strict limitations on valid designs (limited to 54 unique patterns)

By limiting the number of valid designs, the task becomes increasingly difficult as contestants exhaust easier options. This encourages strategic problem solving, but also increases the likelihood of errors (rule violations and repetitions), providing a more sensitive measure of executive function and cognitive flexibility.

##### 2.3.1.1 Task rules and valid designs

Participants must create designs that meet the following constraints:

- Lines must connect only nearest neighbors (diagonal connections are not allowed).
- Designs may use two, three, or four dots, but not all five.
- Only one continuous line is allowed per design.
- Designs must have a clear start and end point (T-shaped or closed-loop designs are not allowed).

A complete list of all 54 valid designs, ordered by complexity, and detailed task instructions are provided in Appendix 2.

##### 2.3.1.2 Instructions

The examiner sits across from the participant, who is given the test sheet with a movable 3 × 3 cm window that guides the order of completion. The contestant has 5 min to create unique designs while following specific rules. The timer runs continuously. At 3 min, the participant is reminded of the remaining time (“2 min left”) and again at 4 min (“1 min left”). If a participant is having trouble coming up with a new design, he or she is encouraged non-specifically (e.g., “You still have time-maybe you can come up with another idea”), but no explicit suggestions are given.

Errors are corrected only once within the first five designs (e.g., if a participant repeats a pattern early on, they are informed once). All subsequent errors remain uncorrected.

Clearly structured instructions with demonstrations are given before starting. For example:

“Look at this test sheet (cf. Appendix 2)—each small box contains five dots arranged in the same way. Your task is to connect two, three, or four nearest dots with a single straight line to create as many different designs as possible. No two patterns can be the same. Let me show you an example. (Examiner demonstrates by drawing a simple valid pattern.)”

“There are also some important rules: You cannot connect all five dots, your design must be a continuous line with only one starting and ending point, and you cannot see your previous designs-so you must remember them. I will guide you with this sliding window to help you focus on one box at a time. You have 5 min to create as many different designs as you can. Are you ready? Let us get started!”

##### 2.3.1.3 Scoring

1. Productivity: The number of unique, valid designs produced, expressed as a percentage of the maximum possible (54).
2. Accuracy:
  Rule Violations: Proportion of designs that violated task constraints. This is calculated as the number of rule-breaking designs divided by the total number of designs produced.
    Repetitions: Proportion of designs that were repeated. This is calculated as the number of repeated designs divided by the total number of designs produced.

By analyzing productivity and these two aspects of accuracy separately, the m5PD task provides distinct insights into executive function, particularly cognitive flexibility, inhibitory control, and working memory.

#### 2.3.2 Modified word-fragment completion task

The modified word fragment completion (mWFC) task is a visual, orthographic retrieval task without semantic cues that requires spelling, grammatical classification, and cue-based retrieval. Participants complete C_C trigrams (e.g., R_T) by selecting a vowel from a physical vowel board to form valid German nouns. Responses are immediate and irreversible, increasing sensitivity to errors in lexical access and decision making. Participants have up to 5 min to complete 54 trigrams, stopping when finished or when time runs out. The mWFC task assesses lexical retrieval, orthographic processing, and decision making under interference.

Three major task characteristics were designed to be cognitively demanding:

1. Task length (up to 5 min)

Similar to the m5PD task.

1. Movable display window (previous trigrams were hidden after completion)

Similar to the m5PD task.

1. Task flow

Participants view a C_C trigram and select a vowel from the vowel board, which displays A, I, E, O, U, with Y as a distractor, by pointing to the vowel. Once a vowel is selected, the trigram disappears, preventing revision. The task consists of 54 trigrams, including seven ambiguous trigrams that appear twice to assess response consistency (e.g., the trigram ‘H_F’, which can be completed as ‘HOF’ (yard) or ‘HUF’ (hoof), appeared twice).

A complete list of all 54 trigrams and detailed task instructions are provided in Appendix 3.

##### 2.3.2.1 Instructions

The examiner sits facing the patient, with the test sheet placed centrally in front of him or her. The vowel board should be placed on the unaffected side of the patient - on the right for those with right hemisphere infarcts and on the left for those with left hemisphere infarcts. This ensures that the patient can select a vowel with the unaffected hand.

The task lasts exactly 5 min, during which the timer runs continuously. After 3 min, the examiner announces, “You have 2 min left,” and after 4 min, “You have 1 min left.

The goal is to insert a single vowel between two consonants to form a proper noun. The letter “Y” does not count as a vowel. For example, given the consonants “B” and “R,” a valid answer would be “BAR.” However, repeating the same word in another box is not allowed, so a different vowel must be chosen each time (e.g., “BOR” for the chemical substance). Certain answers are not allowed: articles like “DER,” names like “ROM,” English words like “CAT,” and abbreviations like “KAT.”

Once the timer starts, the examiner moves through the test using a sliding window, advancing only when the patient has completed the current box. If the patient gets stuck, he or she should select any vowel to continue. No specific hints are allowed, but general encouragement such as “You still have time-maybe another idea will come to you” is permitted.

Errors are corrected only once, and only if they occur within the first five boxes. If a patient makes their first error after this point, no corrections are given.

Before starting, the examiner briefly explains the task and asks if the patient has any questions. When they are ready, they say “Go!” and start the five-minute timer.

##### 2.3.2.2 Scoring

Performance is measured using:

Productivity: Number of correct answers divided by 54.
Accuracy: Number of incorrect responses divided by the number of total responses (excluding the very rare “Y” choices).

Incorrect answers can occur for many reasons:

Lexical misidentification - Choosing a vowel that does not form a valid noun. Non-word errors - [e.g., “RET” or “RIT” instead of “RAT” (advice)].
Similar word interference - Choosing a completion because it competes with more common alternatives. Grammatical category error (non-noun errors) - Choosing a vowel that does not make a valid German noun (e.g., “ROT” (red) instead of “RAT”). Non-linguistic errors (non-word errors) - Choosing a vowel to form a proper noun (e.g., “RUT” as a name) or an acronym.
Impulsive selection - The need for a quick response increases the likelihood of errors.
Carry-over interference - Repetition of previous vowel choices.
Distractor interference - Previously used vowels and the presence of the “Y” increase the likelihood of selection errors.

Unlike the m5PD task, where errors result from either rule violations or design repetitions, errors in the mWFC task result from incorrect lexical decisions rather than predefined constraints. Valid error classification is not possible in the mWFC task because the five available vowels must be reused across 54 trigrams, making it impossible to distinguish between repetitions and otherwise invalid responses. Some trigrams appeared twice, adding further ambiguity - choosing the same vowel on both occasions may reflect a repetition error, but the number of trigram repetitions was insufficient to reliably assess repetition propensity. Because vowel choices are dictated by lexical validity rather than task rules, all incorrect responses (non-words, non-nouns, or non-linguistic errors) are combined into a single accuracy measure, as opposed to the more precise error classification possible in the m5PD task.

Thus, the m5PD and mWFC tasks differ in their error specificity due to task constraints. The m5PD task separates rule violations (breaking task constraints) from repetitions (reusing previous designs), allowing detailed analysis of inhibitory control and proactive interference. The mWFC task uses a general error measure because repetition errors cannot be reliably identified. Thus, all incorrect responses (non-words, non-nouns, or non-linguistic errors) are grouped under a single accuracy measure.

The m5PD and mWFC tasks were selected to assess different cognitive domains - visuospatial and verbal fluency - while measuring both productivity and accuracy within each domain. The m5PD task assesses spatial flexibility by requiring participants to generate unique designs under strict rule constraints. In contrast, the mWFC task measures lexical access and orthographic processing through immediate vowel selection in word fragment completion. Accuracy is assessed more specifically in the visuospatial domain, where rule violations and repetitions can be distinguished, whereas in the verbal domain all incorrect responses are combined into a single error measure due to the inherent constraints of vowel selection. These differences make the two tasks complementary in assessing domain-specific facets of cognitive productivity and the maintenance of accurate task performance.

### 2.4 Psychometric analysis

Chapman and Chapman (1973) in considering problems of measuring cognitive deficits, have long ago argued that psychometrically matched behavioral measures are essential for meaningful comparisons, ensuring that each measure within a study is equivalent in difficulty, reliability, and validity. Psychometric matching allows differences in performance to be attributed to inherent, rather than psychometric, differences between the measures. Although the design of psychometrically matched measures is challenging and requires extensive calibration, it is critical to the integrity of many study designs. Failure to use appropriately matched behavioral measures can undermine the validity of voxel-based lesion mapping and lead to misleading conclusions. Appendix 1 presents the results of a pilot study conducted to calibrate the reliability and validity of the study measures. As a correlative method, lesion mapping is limited by the reliability of the measures used because unreliable measures introduce more measurement error, resulting in attenuated correlations. Consequently, the validity of lesion mapping relies on the use of behavioral measures with matched reliability.

**Figure 1:**
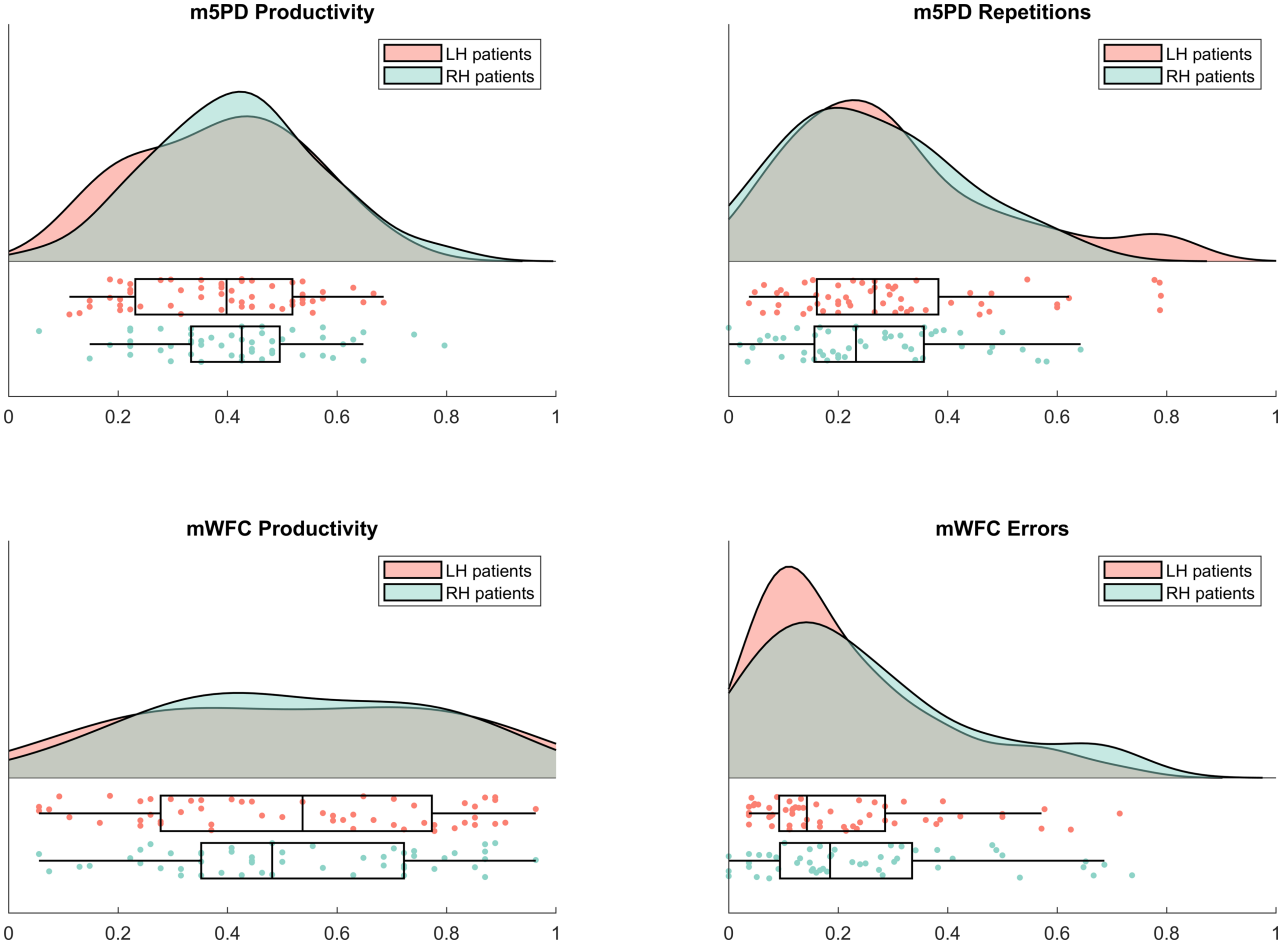
Raincloud plots of the behavioral measures. The sample distributions of m5PD (top panels) and mWFC (bottom panels) productivity (left panels) and accuracy (right panels). Data points represent individual scores overlaid by box plots showing the sample median and interquartile range. The raincloud plots were generated using codes provided by Allen et al. (2019).

Therefore, we were concerned with ensuring sufficient and comparable reliability (in terms of internal consistency) of the behavioral measures. We estimated their split-half reliabilities using the sampling method of Steinke and Kopp (2020). The resulting reliability estimates were 0.90 (m5PD productivity), 0.97 (mWFC productivity), 0.91 (m5PD accuracy), and 0.86 (mWFC accuracy). These coefficients indicate good reliability for all behavioral measures considered (all estimates > 0.85). At most, there are minor differences between these reliability estimates, which, if they exist, may reflect the distributional properties of the behavioral measures shown in Figure 1. Overall, the results of this analysis suggest that the use of the four behavioral measures together in a study mapping behavioral symptoms to brain lesions is justified on these psychometric grounds.

### 2.5 Lesion mapping

Structural brain images of all patients were acquired on two 3 T MRI scanners (Siemens Magnetom Verio and Magnetom Skyra, Siemens Healthineers, Erlangen, Germany) and two 1.5 T MRI scanners (Siemens Magnetom Avanto and Magnetom Aera, Siemens Healthineers, Erlangen, Germany) at the Institute of Diagnostic and Interventional Neuroradiology, Hannover Medical School, under the direction of HL. Information on imaging acquisition variables is provided in Appendix 4, for each scanner model.

For each patient, a lesion mask was manually delineated on diffusion-weighted magnetic resonance imaging (DW-MRI) obtained at b1000 or b1500—images highly sensitive for identifying (sub-)acute ischemic strokes. This was achieved using MRIcron.^1^ The validity and accuracy of the traced lesion masks were jointly monitored and validated by the senior neurologist, KW, and the expert neuroradiologist, MD.

Lesion masks were normalized using the Clinical Toolbox (Rorden et al., 2012) with enantiomorphic normalization (Nachev et al., 2008). Prior to normalization, each lesion mask, initially delineated on high b-value DWI, was co-registered to the native-space DWI B0 image of the same patient using SPM’s affine transformation. This step ensures accurate alignment between the clinically-derived lesion mask and the B0 image, which provides better anatomical contrast for subsequent normalization. The co-registered lesion and B0 images were then subjected to the normalization process, which used an age-appropriate template provided in the Clinical Toolbox for SPM (Rorden et al., 2012) to ensure anatomical compatibility with our patient population, and applied both affine and nonlinear spatial transformations to ensure optimal anatomical alignment. During normalization, lesioned voxels were replaced with their contralateral counterparts prior to spatial transformation to reduce warping artifacts and preserve structural integrity. The following parameters were used: Bounding box = [−78–112 − 50; 78 76 85], voxel size = 1 × 1 × 1 mm^3^.

Identification of brain regions associated with performance on the m5PD (spatial fluency) and mWFC (verbal completion) tasks was performed using multivariate voxel-based lesion symptom mapping (VLSM) with sparse canonical correlation analysis (SCCAN; Pustina et al., 2018), implemented in the LESYMAP package^2^ in R.^3^ This optimization approach determines the optimal weights to apply to each voxel to identify the pattern of brain lesions that has the strongest statistical association with the observed behavioral scores. The sparseness value, which controls the proportion of voxels retained in the final solution, was determined by the standard cross-validation (CV) routine. The goodness of the final VLSM solution and its statistical significance were assessed by calculating the CV correlation between the observed and predicted behavioral scores. Prior to analysis, all behavioral measures, which are proportions, were transformed using the arcsine square root method.

**Figure 2:**
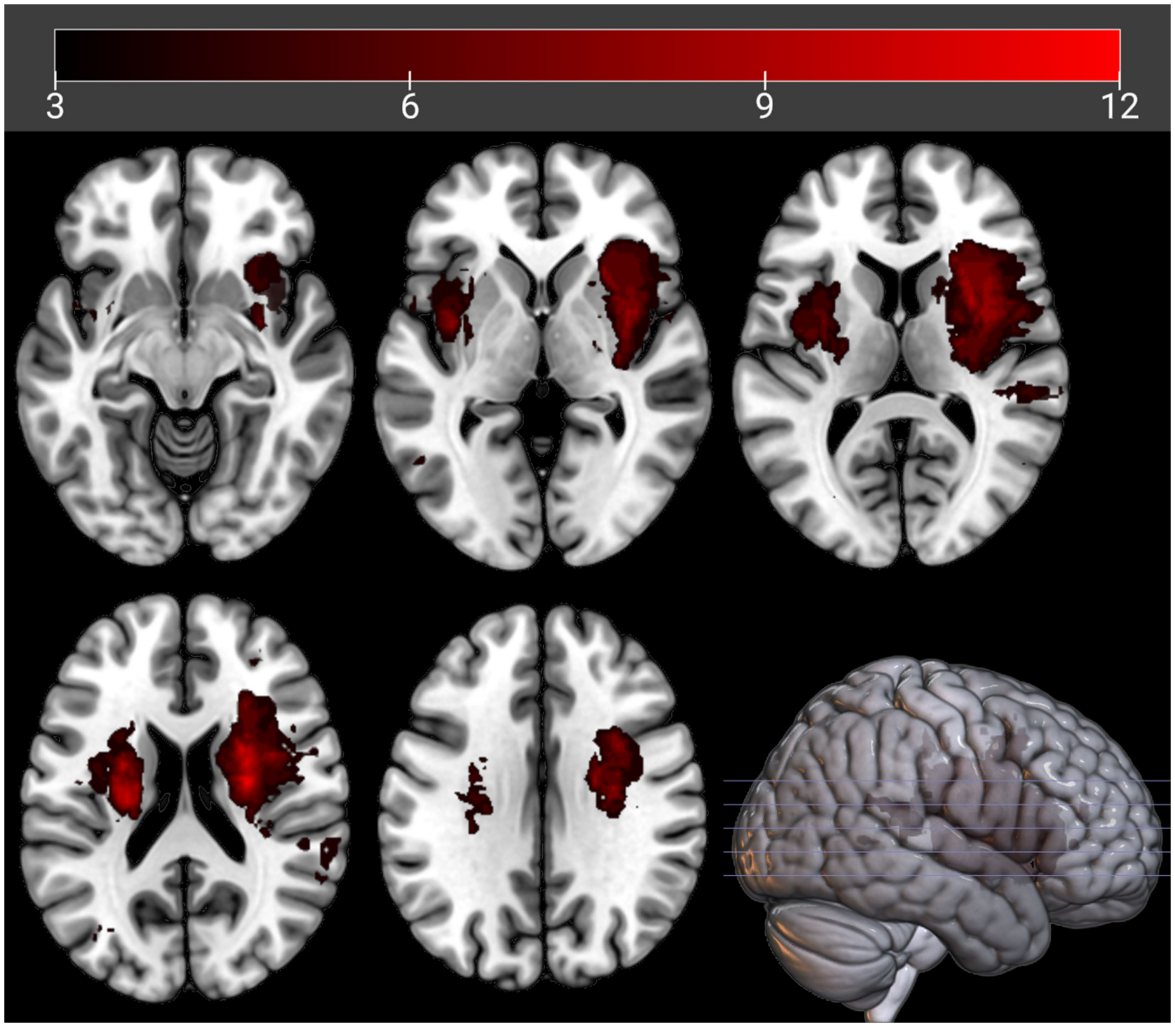
Lesion coverage. The maps show the overlap of participants’ lesions voxel-wise, with a minimum of three participants’ lesions in each voxel and a maximum of 12 participants’ lesions in each voxel. Left/right orientation convention in which the left side of the image corresponds to the left side of the patient.

We included only voxels lesioned in at least three patients (see Figure 2), consistent with prior studies using the SCCAN method in LESYMAP (Tarantino et al., 2022). Given the limited lesion overlap in our dataset (maximum 12 patients per voxel), this threshold balances statistical power with spatial coverage, ensuring robust and reliable results while minimizing noise from sparsely lesioned voxels.

The primary analyses were conducted without covarying for demographics, i.e., age, sex, and years of education, while supplementary analyses were performed to assess their potential influence (see Appendix 5).

To explore the probabilistic disconnection patterns associated with SCCAN results, the binary maps of SCCAN suprathreshold voxels were mapped onto an atlas of white matter tracts (Rojkova et al., 2016) using the TRACTOTRON method (Foulon et al., 2018) in BCBtoolkit.^4^ Specifically, TRACTOTRON returns the probability of structural disconnection caused by a focal lesion for each of the 68 tracts present in the atlas (see also Tarantino et al., 2022). We classified the likelihood of tract disconnection as follows: high (probability > 0.8), moderate (0.5 < probability < 0.8), and negligible (probability < 0.5). These thresholds were chosen to provide a qualitative interpretation of the TRACTOTRON output.

## 3 Results

### 3.1 Behavioral results on neuropsychological screenings

Table 1 shows the neuropsychological screening data of the study patients. Inspection reveals that the sample consisted of predominantly right-handed patients who were cognitively relatively well-functioning (in terms of language and neglect symptoms), as intended, with most of them without severe symptoms of depression at the time of assessment.

### 3.2 Behavioral results on the two generative tasks

Table 2 shows the behavioral results of the modified five-point design (m5PD) fluency task. On average, patients produced valid designs with a mean productivity of 40% (approximately 22 valid productions out of 54 possible), a median of 41%, and a range of 6% (three valid designs) to 80% (43 valid designs). Rule breaks were rare, with a mean error rate of 11%, a median of only 6%, and a range of 0 to 79%. Repetitions were more common, with a mean error rate of 29%, a median of 26%, and a range of 0 to 79%. These results suggest that patients were generally adept at producing valid designs and committed fewer rule breaks than repetitions. There was considerable inter-individual variability in all aspects of the task, highlighting the wide variation in patient performance.

**Table 2:** Summary of the behavioral measures (in percentage terms).

	M	SD	Med	Min	Max
m5PD task					
Productivity	40	15	41	6	80
LHem (*N* = 56)	39	15	40	11	69
RHem (*N* = 51)	42	15	43	6	80
Rule breaks	11	14	6	0	79
LHem (*N* = 56)	9	11	4	0	46
RHem (*N* = 51)	13	16	6	0	79
Repetitions	29	18	26	0	79
LHem (*N* = 56)	30	20	27	4	79
RHem (*N* = 51)	26	16	23	0	64
mWFC task					
Productivity	52	26	50	6	96
LHem (*N* = 55)	52	27	54	6	96
RHem (*N* = 51)	53	24	48	6	96
Accuracy	23	18	17	0	74
LHem (*N* = 55)	21	17	14	4	71
RHem (*N* = 51)	25	20	19	0	74

Productivity is measured as the percentage of valid productions relative to the maximum number of productions possible on each task, which is 54. Error rates are percentage measures relative to the number of actual productions generated for each task. For example, if 27 valid items were generated on a task, the corresponding productivity measure would be 50 percent (27/54 = 0.50). If there were also 9 errors, the corresponding error rate would be 25 percent (9/(27 + 9) = 0.25).

In the remainder of the analysis, rule breaks were excluded. Their infrequency affects their reliability, making their measurement quality less satisfactory than repetitions, which were more frequent. The m5PD accuracy variable refers only to the number of repetitions of the m5PD task.

Table 2 also shows that on the mWFC task, patients produced valid vowels with a mean productivity of 52% (approximately 28 out of 54), a median of 50%, and a range of 6% (three correct vowels) to 96% (52 correct vowels). Errors were common, with a mean of 23%, a median of 17%, and a range of 0 to 74%. These results indicate that patients were generally adept at producing correct vowels, but also made a significant number of errors. Again, there was considerable variability in performance between patients.

Descriptive statistics for the productivity measure (Table 2) show that the means for the m5PD task are 0.39 (*SD* = 0.15) for left lateralized lesions and 0.42 (*SD* = 0.15) for right lesion laterality. For the mWFC task, the means are 0.52 (*SD* = 0.27) for left laterality and 0.53 (*SD* = 0.24) for right laterality. For the accuracy measure, the means for the m5PD task are 0.30 (*SD* = 0.20) for left laterality and 0.26 (*SD* = 0.16) for right laterality. For the mWFC task, the means are 0.21 (*SD* = 0.17) for left laterality and 0.25 (*SD* = 0.20) for right laterality. The repeated measures ANOVA shows significant within-subject effects for Measure, Task, and their interaction. Measure has *F*(1, 104) = 54.25, *p* < 0.001; Task has *F*(1, 104) = 9.91, *p* = 0.002; and their interaction has *F*(1, 104) = 19.24, *p* < 0.001. The interactions between Measure and Laterality, Task and Laterality, and Measure, Task, and Laterality are not significant (*p* values of 0.824, 0.286, and 0.271). Between-subjects effects indicate that lesion laterality is not significant (*F*(1, 104) = 0.50, *p* = 0.480). These results show differences in means for measure and task, but variations in lesion laterality do not significantly affect the results.

Figure 1 shows raincloud plots of all the behavioral measures considered in the lesion mapping analysis, with productivity measures in the left panels, accuracy measures in the right panels, data from the m5PD task in the top panels, and data from the mWFC task in the bottom panels. The plotted distributions show that the m5PD and mWFC productivity distributions are approximately symmetric with medians close to 0.5, with unimodal and bimodal distributions, respectively. In contrast, the accuracy measures are right-skewed with medians around 0.25, indicating relatively few errors overall, but some patients with higher error rates.

### 3.3 Lesion coverage

Figure 2 shows the spatial lesion overlap for all 110 patients studied. The lesion overlay maps highlight brain regions affected in at least three patients per voxel, consistent with the limitations of multivariate VLSM based on SCCAN, and outline those brain regions for which conclusions can be drawn. The greatest overlap is in the periventricular white matter in both hemispheres. In the cortex, the highest lesion overlap is in the right inferior frontal cortex and insula.

Most patients had lesions in the unilateral (either left or right) frontal cortex and underlying white matter. Stroke lesions in frontal regions can result from different types of stroke, depending on the vascular territory involved. The major types include strokes involving specific divisions of the middle cerebral artery (MCA), anterior cerebral artery (ACA) strokes, ACA-MCA border zone (watershed) infarcts, and lacunar strokes (e.g., lentiform nucleus infarcts). Only patients with large MCA strokes resulting in extensive cortical lesions in the perisylvian areas were excluded because they are typically associated with severe symptoms of aphasia or hemi-neglect (see Section 2.2 for specific exclusion criteria).

To characterize the anatomical distribution of lesions in individual patients, we used the Harvard-Oxford cortical and subcortical atlases (Desikan et al., 2006) and quantified the number of patients with lesions in each anatomical region, distinguishing between left and right hemispheres. This analysis provided a comprehensive overview of lesion distribution across cortical and subcortical structures. The complete distribution of lesions in all cortical and subcortical regions is shown in Table 3.

**Table 3:** The anatomical distribution of lesions in individual patients.

Region	Left hemisphere	Right hemisphere
Frontal Pole	15	9
Insular Cortex	30	24
Superior Frontal Gyrus	12	20
Middle Frontal Gyrus	26	19
Inferior Frontal Gyrus pars triangularis	16	10
Inferior Frontal Gyrus pars opercularis	16	8
Precentral Gyrus	37	30
Temporal Pole	8	9
Superior Temporal Gyrus anterior division	5	5
Superior Temporal Gyrus posterior division	9	6
Middle Temporal Gyrus anterior division	3	2
Middle Temporal Gyrus posterior division	8	6
Middle Temporal Gyrus temporooccipital part	12	11
Inferior Temporal Gyrus anterior division	1	0
Inferior Temporal Gyrus posterior division	5	4
Inferior Temporal Gyrus temporooccipital part	5	8
Postcentral Gyrus	29	28
Superior Parietal Lobule	13	7
Supramarginal Gyrus anterior division	11	6
Supramarginal Gyrus posterior division	15	10
Angular Gyrus	13	11
Lateral Occipital Cortex superior division	17	15
Lateral Occipital Cortex inferior division	11	11
Intracalcarine Cortex	3	6
Frontal Medial Cortex	1	0
Supplementary Motor Cortex	4	2
Subcallosal Cortex	2	0
Paracingulate Gyrus	3	5
Cingulate Gyrus anterior division	3	10
Cingulate Gyrus posterior division	3	6
Precuneus Cortex	6	9
Cuneal Cortex	0	7
Frontal Orbital Cortex	16	8
Parahippocampal Gyrus anterior division	5	7
Parahippocampal Gyrus posterior division	0	2
Lingual Gyrus	2	4
Temporal Fusiform Cortex anterior division	0	1
Temporal Fusiform Cortex posterior division	2	4
Temporal Occipital Fusiform Cortex	1	2
Occipital Fusiform Gyrus	4	4
Frontal Operculum Cortex	16	5
Central Opercular Cortex	28	17
Parietal Operculum Cortex	16	8
Planum Polare	8	7
Heschls Gyrus	8	7
Planum Temporale	11	11
Supracalcarine Cortex	0	4
Occipital Pole	3	7
Cerebral White Matter	59	54
Thalamus	8	6
Caudate	11	15
Putamen	18	26
Pallidum	7	11
Hippocampus	1	1
Amygdala	6	6
Accumbens	0	3

### 3.4 Lesion mapping

#### 3.4.1 SCCAN for the m5PD task (spatial fluency)

**Figure 3:**
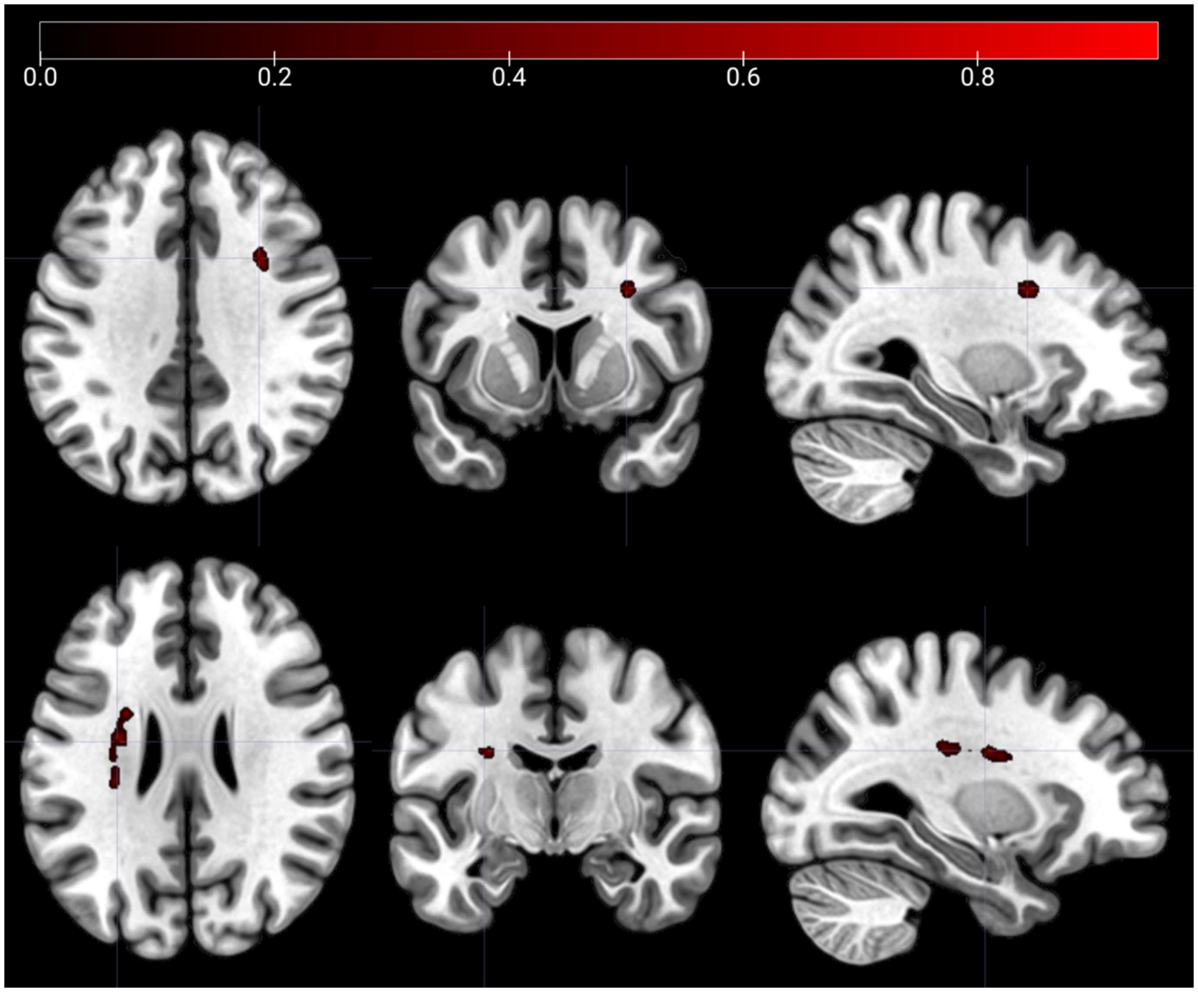
Lesion clusters on the m5PD task. The figure shows lesion clusters obtained from SCCAN that were significantly associated with m5PD accuracy (CV correlation = 0.36, *p* < 0.001). The MNI coordinates of the cluster peaks were: −29, −9, 26 (the lesion cluster shown in the top panels) and 30,9,35 (the lesion cluster shown in the bottom panels). The color map shows the normalized (−1 to 1) voxel weights, which represent the relative importance of each voxel in the multivariate prediction model. The further the weight is from 0, the greater the contribution of that voxel to the lesion pattern that best predicts the accuracy score. Both lesion clusters were positively associated with error rates in the m5PD task, i.e., the presence of a lesion predicts higher m5PD error rates. Left/right orientation convention in which the left side of the image corresponds to the left side of the brain.

The SCCAN VLSM results showed no significant associations between lesions and m5PD productivity (CV correlation = 0.14, *p* = 0.145). However, a significant association was found between lesions and m5PD accuracy (CV correlation = 0.36, *p* < 0.001). Figure 3 identifies two significant lesion clusters: one around Montreal Neurological Institute (MNI) coordinate (30, 9, 35) located in the right inferior frontal operculum, and the other around MNI coordinate (−29, −9, 26) located in the left white matter near the insula.

Table 4 shows SCCAN VLSM results after correction for lesion volume or task productivity. With volume correction alone, lesion locations are consistent with those found without corrections. With productivity correction alone, lesions are seen in the right inferior frontal operculum (MNI: 30, 8, 34), left white matter near the insula (MNI: −31, −25, 27), and near the left caudate (MNI: −28, −3, 26). No specific lesion locations were identified when both corrections were applied. Overall, lesions in the right inferior frontal operculum and left white matter near the insula are consistently associated with m5PD accuracy, with additional involvement of the left caudate when the productivity correction is applied.

**Table 4:** MNI coordinates and AAL anatomical descriptors for lesions associated with accuracy on the m5PD task (spatial fluency) and the mWFC task (verbal completion) without and with correction(s), i.e., yes/no f or lesion volume and productivity on each task.

Task	Measure	Corrections		MNI			AAL anatomy	
		Volume	Productivity	x	y	z	Label	Distance
m5PD	Accuracy	No	No	**30**	**9**	**35**	**Frontal_Inf_Oper_R**	**0,0**
				*−29*	*−9*	*26*	*Insula_L*	*6,2*
m5PD	Accuracy	Yes	No	**30**	**9**	**35**	**Frontal_Inf_Oper_R**	**0,0**
				*−29*	*−9*	*26*	*Insula_L*	*6,2*
m5PD	Accuracy	No	Yes	**30**	**8**	**34**	**Frontal_Inf_Oper_R**	**0,0**
				*−31*	*−25*	*27*	*Insula_L*	*3,6*
				*−28*	*−3*	*26*	*Caudate_L*	*7,5*
m5PD	Accuracy	Yes	Yes	–	–	–	–	–
mWFC	Accuracy	Yes	Yes	**33**	**7**	**28**	**Frontal_Inf_Oper_R**	**1,0**
				**39**	**13**	**9**	**Frontal_Inf_Oper_R**	**0,0**
				*30*	*−10*	*29*	*Insula_R*	*6,6*
mWFC	Accuracy	No	No	**32**	**7**	**29**	**Frontal_Inf_Oper_R**	**0,0**
				*31*	*−10*	*26*	*Insula_R*	*4,1*
mWFC	Accuracy	Yes	No	–	–	–	–	–
mWFC	Accuracy	No	Yes	**33**	**7**	**28**	**Frontal_Inf_Oper_R**	**1,0**
				**39**	**13**	**8**	**Frontal_Inf_Oper_R**	**0,0**
				*30*	*−10*	*29*	*Insula_R*	*6,6*

Common lesion clusters in highlighted in bold. Distinct lesion clusters appear in italics.

#### 3.4.2 SCCAN for the mWFC task (verbal completion)

**Figure 4:**
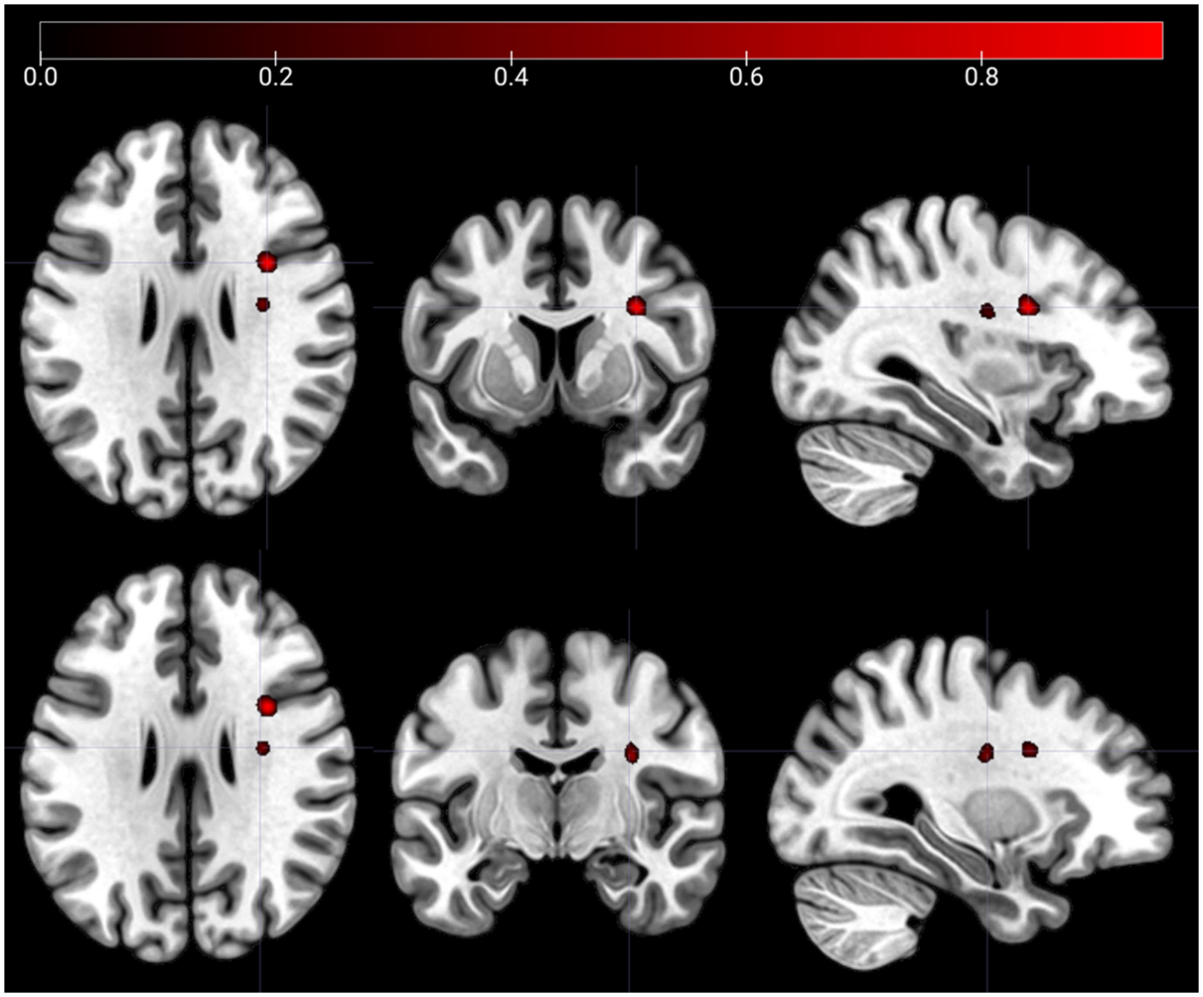
Lesion clusters on the mWFC task. The figure shows lesion clusters obtained from SCCAN that were significantly associated with mWFC accuracy (CV correlation = 0.31, *p* = 0.001). The MNI coordinates of the cluster peaks were: 33,7,28 (the lesion cluster shown in the top panels) and 30,-10,29 (the lesion cluster shown in the bottom panels). The color map shows the normalized (−1 to 1) voxel weights, which represent the relative importance of each voxel in the multivariate prediction model. The further the weight is from 0, the greater the contribution of that voxel to the lesion pattern that best predicts the accuracy score. Both lesion clusters were positively associated with error rates in the mWFC task, i.e., the presence of a lesion predicts higher mWFC error rates. Left/right orientation convention in which the left side of the image corresponds to the left side of the brain.

The SCCAN VLSM results showed no significant associations between lesions and mWFC productivity (CV correlation = 0.17, *p* = 0.085), but a significant association with mWFC accuracy (CV correlation = 0.31, *p* = 0.001). Figure 4 shows significant lesion clusters in the right hemisphere: one around MNI coordinate (33, 7, 28) located in the right inferior frontal operculum and another around MNI coordinate (30, −10, 29) located in the right white matter near the insula.

Table 3 shows the SCCAN VLSM results after correction for lesion volume or task productivity. With volume correction alone, no specific lesions were identified. With productivity correction alone, lesions were found in the right inferior frontal operculum at (MNI: 33, 7, 28 and 39, 13, 8) and above the right insula (MNI: 30, −10, 29). These locations remained consistent when both corrections were applied. Overall, lesions in the right inferior frontal operculum and right insula are consistently associated with mWFC accuracy, especially with the productivity correction, while the absence of specific lesions with the volume correction suggests that lesion volume may be a confounding factor.

In summary, we identified common and distinct lesion correlates of cognitive accuracy, but not cognitive productivity. The accuracy-related common lesion cluster was centered around the right inferior frontal operculum, whereas distinct clusters in frontal white matter showed domain-incongruent hemispheric lateralization of cognitive accuracy.

**Figure 5:**
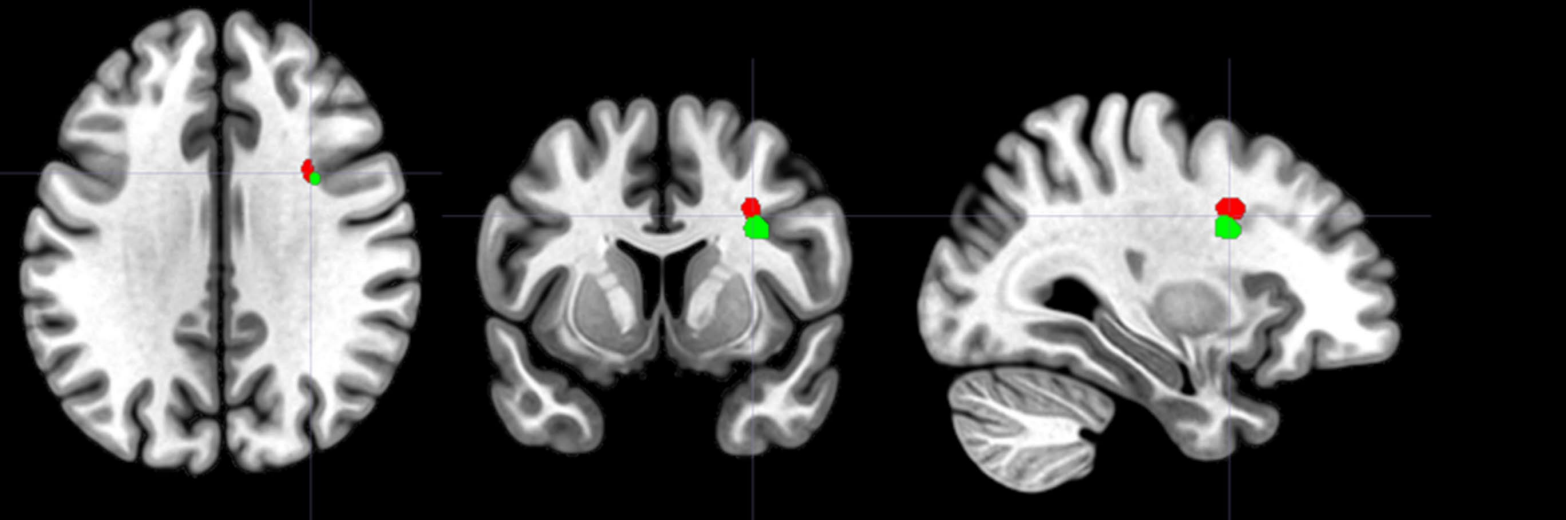
Shared lesion correlates of cognitive accuracy on the m5PD and mWFC tasks. The figure shows lesion clusters in the right inferior frontal operculum that were significantly associated with error rates on the 5PD task assessing spatial fluency (shown in red; cluster peak: 30,9,35) and on the mWFC task assessing verbal completion (shown in green; cluster peak: 33,7,28). Left/right orientation convention in which the left side of the image corresponds to the left side of the brain.

These findings indicate that the right inferior frontal operculum is critical for maintaining cognitive accuracy in both of our tasks, suggesting a domain-general role for this brain region (Figure 5). The two common MNI coordinates, (30, 9, 35) and (33, 7, 28), both correspond to the right inferior frontal cortex according to several anatomical atlases. The AAL atlas (Tzourio-Mazoyer et al., 2002) identifies both coordinates as being in the right inferior frontal operculum. Similarly, the Harvard-Oxford Cortical Structural Atlas (Makris et al., 2006) maps these coordinates to the right inferior frontal gyrus, pars opercularis. According to the Talairach Atlas (Lancaster et al., 2000), both coordinates correspond to the right inferior frontal gyrus, specifically Brodmann’s area 44. This anatomical consistency across atlases suggests that, despite slight differences in coordinates, both points are located in the same region and are likely to produce similar functional effects when lesions occur there. Thus, these coordinates highlight the critical role of the right inferior frontal cortex in maintaining cognitive accuracy in both tasks.

### 3.5 TRACTOTRON findings

Regarding the different MNI coordinates, cognitive accuracy in spatial fluency is associated with left white matter lesions, whereas verbal completion is associated with right white matter lesions (see Figures 3, 4). To describe the white matter tracts involved, Table 5 shows the probabilities of tract lesions for both tasks. These probabilities indicate the likelihood of each tract’s disconnection given the significant lesions found in the VLSM analysis.

**Table 5:** Probabilistic structural disconnection patterns associated with cognitive accuracy (error rates) on the m5PD (spatial fluency) and mWFC (verbal completion) tasks.

Fiber Tract	m5PD	mWFC
Corpus callosum	**0.93**	**0.84**
Frontal commisural tract	0.66	0.5
Left cerebral hemisphere		
Superior longitudinal fasciculus II	**0.93**	0
Superior longitudinal fasciculus III	**0.99**	0
Arcuate long segment	**0.84**	0
Frontal aslant tract	**1.00**	0
Frontal inferior longitudinal tract	0.57	0
Fronto-striatal tract	**0.98**	0
Fronto-pontine projections	**0.98**	0
Anterior thalamic radiation	**0.92**	0
Corticospinal tract	**1.00**	0
Right cerebral hemisphere		
Superior longitudinal fasciculus II	**0.98**	**0.98**
Superior longitudinal fasciculus III	0.63	**1.00**
Arcuate anterior segment	0.36	**0.96**
Arcuate long segment	0.3	**0.88**
Frontal aslant tract	0.7	**0.94**
Frontal inferior longitudinal tract	0.78	**0.9**
Fronto-insular tract 4	0	0.54
Fronto-insular tract 5	0	0.52
Fronto-striatal tract	0.3	**0.96**
Fronto-pontine projections	0.24	**0.86**
Corticospinal tract	0.61	**1.00**

The TRACTOTRON table shows probabilities *p* > 0.50 (in one hemisphere) of white matter tracts being (partially) disconnected given the significant lesion clusters identified in the SCCAN analyses. The remaining tract probabilities were *p* < 0.50 in both hemispheres. High probabilities appear in bold (threshold value *p* > 0.80).

For spatial fluency (m5PD task), many left hemisphere tracts, such as the superior longitudinal fasciculus (II and III), long arcuate segment, frontal aslant tract, fronto-striatal tract, fronto-pontine projections, anterior thalamic radiation, and corticospinal tract, show high involvement. The right hemisphere is less involved, with high probability only in the superior longitudinal fasciculus II and moderate involvement in the anterior arcuate segment and frontal aslant tract. This suggests extensive left hemisphere involvement in maintaining cognitive accuracy on the m5PD task, with the right hemisphere playing a minor role.

For verbal completion (mWCF task), the left hemisphere shows moderate involvement only in the frontal commissural tract, with other tracts showing negligible involvement. In contrast, many right hemisphere tracts, including the superior longitudinal fasciculus (II and III), arcuate segments, frontal aslant tract, frontal inferior longitudinal tract, fronto-insular tracts, fronto-striatal tract, fronto-pontine projections, and corticospinal tract, show high involvement. This suggests extensive right hemisphere involvement in maintaining cognitive accuracy in the mWFC task, with minimal left hemisphere involvement.

Interhemispheric connections, represented by the corpus callosum and the frontal commissural tract, show high m5PD probabilities and somewhat lower but notable mWFC probabilities, suggesting that structural lesions in these tracts generally affect cognitive accuracy variability. Key tracts such as the superior longitudinal fasciculus, arcuate fasciculus, and frontal aslant tract are critical for both tasks, but with different hemispheric dominance: the left hemisphere is more involved in cognitive accuracy for spatial fluency, whereas the right hemisphere is more involved in verbal completion. The left hemisphere shows high m5PD and zero mWFC probabilities in both short- and long-range tracts, suggesting that widespread left intrahemispheric disconnection affects cognitive accuracy only in the m5PD task. Conversely, the right hemisphere shows more balanced probabilities for both metrics, with stronger evidence for the role of right intrahemispheric connectivity in cognitive accuracy for the mWFC task. Taken together, this study reveals a domain-incongruent hemispheric lateralization: left hemispheric connectivity predominantly supports cognitive accuracy in spatial fluency, whereas right hemispheric connectivity is critical for verbal completion accuracy.

For the sake of clarity, the results of this analysis are intended only to provide an indication of which tracts correspond to the lesion clusters identified in the VLSM analysis, rather than to associate lesions in specific white matter tracts with specific deficits.

## 4 Discussion

This study investigated the neural correlates of cognitive performance in spatial fluency and verbal completion tasks in stroke patients, with a focus on accuracy. Although no significant correlations between productivity and lesion locations were found, our results reveal distinct neural correlates for error measures that support both domain-general central monitoring and a reverse lateralized error-monitoring system. In spatial fluency, lesion clusters in the right frontal operculum and left white matter suggest that these regions contribute to the maintenance of spatial accuracy. In verbal completion, accuracy was associated with lesions in the right frontal operculum and right white matter, implicating these areas in the maintenance of verbal accuracy. Thus, our results suggest a complex pattern of lateralization: a common lesion cluster in the right frontal operculum supports central monitoring across domains, whereas domain-incongruent lateralization of white matter - left for spatial errors and right for verbal errors - is consistent with the error-monitoring hypothesis (Hochman and Eviatar, 2004, 2006). This hypothesis posits an inversely lateralized system, with left hemisphere dominance for spatial error monitoring and right hemisphere dominance for verbal error monitoring, which is consistent with our observed white matter-error correlations.

The common lesion-error correlation observed in the right inferior frontal region is consistent with previous studies (summarized in the Introduction) highlighting its critical role in maintaining accuracy across task domains. Functional neuroimaging studies have implicated the inferior frontal opercular region in domain-general task control, suggesting its involvement in central executive processes, including performance monitoring (Assem et al., 2020; Cai et al., 2024; Dosenbach et al., 2008). Although our results suggest that the right inferior frontal region supports accuracy across domains, they do not imply that central monitoring is exclusively right lateralized. This is an important consideration given that our lesion coverage in the inferior frontal region was lower in the left hemisphere - likely due to exclusion criteria related to aphasia - so a similar role for the left inferior frontal region cannot be ruled out. Furthermore, after controlling for age, sex, and education, the significance of the right inferior frontal cluster for spatial errors was lost, raising the question of whether its contribution to accuracy reflects a truly domain-general, central function.

While the right inferior frontal region may support domain-general accuracy, the observed domain-incongruent white matter lateralization suggests that neural networks for accuracy diverge across task domains and cerebral hemispheres. Specifically, lesions in left white matter were associated with spatial accuracy, whereas lesions in right white matter were associated with verbal accuracy. This reverse lateralization is consistent with the error-monitoring hypothesis, which proposes that the nondominant hemisphere monitors dominant hemisphere processing for errors (Hochman and Eviatar, 2004, 2006). Thus, our results suggest that reverse lateralized error-monitoring processes contribute to the maintenance of accuracy, although overt errors and error monitoring are clearly distinct phenomena. The fact that lateralized lesions did not directly modulate overt spatial or verbal error rates further underscores the multidimensional nature of overt error generation.

Taken together, our results suggest that cognitive accuracy are supported both by a domain-general, possibly right-lateralized network and by domain-incongruently lateralized networks that are widely distributed, as described in detail in the Results. This view is consistent with previous research (Cipolotti et al., 2020; Mandal et al., 2020; Robinson et al., 2021; McCall et al., 2022). However, clinical studies using go/no-go and stop-signal tasks have reported inconsistent lateralization patterns - some finding increased commission errors in right lateral prefrontal patients (Aron et al., 2003; Molenberghs et al., 2009), while others report no effects or increased omission errors (Krämer et al., 2013; Picton et al., 2007). Similarly, task-switching studies have yielded mixed results, with left PFC lesions associated with rule-switching difficulties and right PFC lesions associated with inhibitory deficits (Mayr et al., 2006), while other studies find increased errors with inferior medial frontal lesions (Shallice et al., 2008, with lesions not being clearly confined to either the left or the right hemisphere) or increased switching costs in both hemispheres (Aron et al., 2004). In addition, results from the Trail Making Test, Part B (TMT-B) are mixed, with some studies reporting slower performance and more errors in frontal patients, especially with bilateral lesions (Stuss et al., 2001), while others find no significant differences between frontal and non-frontal patients (Chan et al., 2015, regardless of whether the frontal damage was in the left or right hemisphere). Lesion-behavior mapping studies further implicate both left (Varjacic et al., 2018) and right (Kopp et al., 2015) lesions in TMT-B performance. These inconsistencies are likely due to methodological differences and small sample sizes, highlighting the complexity of delineating the neural bases of cognitive accuracy.

Studies suggest that stroke, neurodegenerative disease, and aging (Hoffmann and Schmitt, 2006; Hämmerer et al., 2011; Kopp et al., 2014; Al Banna et al., 2016; Giannouli and Tsolaki, 2023) are all associated with declines in performance monitoring - as evidenced, for example, by age-related declines in error-related negativity (Falkenstein et al., 2001). However, stroke studies offer the particular advantage of linking focal brain lesions to specific aspects of performance monitoring, as shown in the present study. For example, Hochman et al. (2015) found a double dissociation: damage to the basal ganglia impairs error correction (recognizing and correcting errors), while damage to the dorsomedial prefrontal cortex impairs error inhibition (preventing errors before they occur). This underscores the critical contributions of clinical neuropsychology to our understanding of the relationship between neural structures and behavioral performance. Clinical studies using lesion-behavior mapping clearly provide a complementary method to electrophysiology and functional brain imaging.

While our findings are consistent with the error-monitoring hypothesis, alternative models remain viable. One such model is the cognitive interference hypothesis (Kopp, 2025). It proposes that the left hemisphere controls proactive interference (PI), preventing old information from interfering with new processing. The right hemisphere controls retroactive interference (RI), protecting previously stored information from new input. PI sensitivity, which may be reflected, for example, in the Stroop effect due to the prior availability of semantic information that interferes with the later availability of color information, is associated with the left PFC in both verbal and spatial task versions (Cipolotti et al., 2016; Perret, 1974; Tsuchida and Fellows, 2013; Ambrosini and Vallesi, 2017). RI sensitivity is associated with the right PFC, perhaps as assessed by the Hayling effect, in which later available congruent closing words interfere with earlier prepared incongruent closing words (see Introduction; Volle et al., 2012; Cipolotti et al., 2016). PI may also gradually accumulate in spatial fluency tasks due to their high self-structuring demands, explaining why left hemisphere lesions correlate with spatial repetition errors. Conversely, RI may be more prominent during vowel selection in verbal completion tasks, potentially linking right hemisphere lesions to verbal errors. To disentangle these two models, future research should systematically manipulate PI and RI across domains and examine lesion effects. The error monitoring hypothesis predicts that left lesions will be associated with spatial accuracy and right lesions will be associated with verbal accuracy, regardless of the type of interference, whereas the interference hypothesis predicts that left lesions will be associated with PI-enriched accuracy and right lesions will be associated with RI-enriched accuracy, regardless of the domain.

## 5 Study limitations and future directions

Among the most significant confounds is the high-dimensional nature of the differences between our spatial fluency and verbal completion tasks. Although we designed these tasks to be both structurally parallel and highly reliable, achieving comparable validity has been more challenging due to inherent differences beyond the intended domain distinctions. A key factor is the self-structuring demands of each task, an essential aspect of assessing executive functioning (Lezak, 1995). Spatial fluency places high demands on self-structuring, requiring participants to plan and organize unique designs in a self-ordered sequence, adding complexity beyond mere production. In contrast, verbal completion involves minimal self-structuring, which may reduce its sensitivity to executive functions. Another important difference is the measurement of errors: spatial fluency allows for a more nuanced classification of errors (i.e., repetitions, rule violations), whereas verbal completion lacks this granularity, introducing an additional confound when comparing performance accuracy across tasks.

Our study distinguishes between productivity and accuracy measures. It also addresses a common limitation of previous research, namely the use of tasks that are too easy, often resulting in low error rates (see Introduction). By increasing task difficulty, we ensured consistently high error rates on both tasks (~25 percent), resulting in similarly reliable accuracy measures (see Methods). However, productivity and accuracy differ in their validity. Productivity, defined as the percentage of items completed, is a relatively process impure measure. It is affected by confounding factors such as overall processing speed, including sensory and motor speed, which can be related to stroke, and task engagement. In contrast, error rate is a more process pure measure because it is calculated as the ratio of two behaviorally derived measures: incorrect responses (numerator) divided by total responses (denominator). This ratio (error rate = incorrect responses/total responses) inherently controls for individual differences in such confounding factors and better isolates the cognitive process of interest: monitoring. In support of this argument, our pilot study found that while productivity did not correlate with standard neuropsychological measures, accuracy did show meaningful associations: spatial accuracy correlated with phonemic fluency, again suggesting left hemisphere involvement, while verbal accuracy correlated with visuoconstructive abilities, again suggesting right hemisphere involvement (see Appendix 1). These considerations highlight the importance of further refining performance measures in future studies to increase the specificity of cognitive assessments.

A note of caution regarding white matter disconnection and the limitations of voxel-level lesion mapping seems to be in order. The goal of our analysis was to provide an initial indication of which white matter tracts correspond to the lesion clusters identified by SCCAN, rather than to perform a detailed tract-level lesion mapping analysis aimed at associating specific tracts with cognitive deficits. Our approach superimposes the significant voxel clusters on a white matter atlas to provide general insight into the potential involvement of tracts, but is not intended to establish definitive tract-behavior relationships. However, our goal here was to provide a first step toward understanding the broader anatomical correlates of cognitive accuracy in production tasks, and future studies may benefit from more specific tract-level analyses.

Although lesion location was the primary focus, supplementary analyses showed that covarying for basic demographics provided additional insight into lesion-deficit relationships (see Appendix 5). Specifically, while the initially reported right inferior frontal cluster associated with accuracy in the spatial fluency task did not remain significant after controlling for age, sex, and education, the left frontal white matter cluster showed a robust and significant association with spatial fluency accuracy. These findings highlight the importance of controlling for demographic factors when interpreting lesion-deficit relationships and suggest that the left frontal white matter cluster may play a particularly critical role in spatial fluency. The common brain regions involved in performance monitoring across tasks remain intriguing and warrant further investigation.

Two methodological decisions regarding patient recruitment deserve brief comment. First, we excluded patients with large cortical lesions to minimize confounding variability from individuals who may be unable to perform the production tasks. Second, although our simple neglect measure may not detect subtle or complex forms of neglect, undiagnosed neglect cannot account for our overall findings. Furthermore, excluding the one patient with neglect would not meaningfully alter the results, as this individual’s performance was not a critical outlier. Consequently, asymmetry-related measures were not included as covariates in our analysis.

Despite its limitations, this study provides a basis for further investigation of the neural mechanisms underlying accuracy maintenance using clinical neuropsychological methods. The sample size of 110 patients, while substantial, limits generalizability and underscores the need for replication with larger samples to increase statistical power and broader applicability. Other limitations include task validity and lesion coverage, which warrant further improvements, as discussed above.

## 6 Conclusion

Our study identifies a multidimensional neural architecture for performance monitoring that blends domain-general and domain-specific processes. A central, domain-general monitoring system - anchored at least in the right inferior frontal region - appears to be associated with accuracy across cognitive domains. In addition, we found robust evidence for a distinct, reverse-lateralized monitoring system: lesions in the left hemisphere were associated with spatial accuracy, whereas lesions in the right hemisphere were associated with verbal accuracy. These findings go beyond traditional lateralization models and suggest more complex hemispheric interactions in performance monitoring. If replicated and validated, these findings could advance our understanding of how the human brain organizes performance monitoring and ultimately lead to targeted rehabilitation strategies for stroke survivors.

## Data Availability

The raw data supporting the conclusions of this article will be made available by the authors, without undue reservation.

## Footnotes

^1^ https://people.cas.sc.edu/rorden/mricron/index.html

^2^ https://github.com/dorianps/LESYMAP

^3^ http://www.R-project.org

^4^ http://toolkit.bcblab.com/
