# Supplementary material for: Post-stroke lesion correlates of errors in verbal and spatial production tasks
Source: Front Psychol. 2025 Apr 28;16:1517876. doi: 10.3389/fpsyg.2025.1517876 (PMC12066591; doi:10.3389/fpsyg.2025.1517876)
Supplement: Supplementary file 1 [file Supplementary_file_1.docx]

Post-stroke lesion correlates of errors
in verbal and spatial production tasks

**Antonino Visalli^1,2^, Natasha Maldonado^1^, Mete Dadak^3,4^,
Heinrich Lanfermann^3^, Karin Weißenborn^1^, Bruno Kopp^1*^**

^1^Department of Neurology, Hannover Medical School, Hannover, Germany

^2^Department of Biomedical, Metabolic and Neuroscience, University of Modena and Reggio Emilia, Reggio Emilia, Italy

^3^Institute of Diagnostic and Interventional Neuroradiology, Hannover Medical School, Hannover, Germany

^4^Department of Diagnostic and Interventional Radiology and Neuroradiology, St. Vincenz-Hospital, Paderborn, Germany

*** Correspondence:**Corresponding Author
kopp.bruno@mh-hannover.de

**Supplementary Information (Appendices)**

**Appendix 1:** Pilot study

*Participants*

We analyzed data from inpatients who were admitted to the Department of Neurology at the Hannover Medical School. The patient sample consisted of 45 (17 female, 28 male) neurological patients with various neurological diseases (23 neurodegenerative diseases, 16 neurovascular diseases, 3 neuroinflammatory diseases, 16 other neurological diagnoses). The number of diagnoses exceeds the number of patients because 13 patients had multiple diagnoses. Patients completed questionnaires on handedness (EHI SF; Veale, 2014) and depression (BDI FS German version; Kliem & Brähler, 2013), the CERAD-Plus neuropsychological test battery (Morris et al., 1988; including the Plus items, i.e., phonemic (letter) fluency and Trails A & B; Aebi, 2002), and our experimental tasks (modified five-point design (m5PD) fluency task, modified word-fragment completion (mWFC) task) for initial psychometric validation. The sociodemographic and neuropsychological characteristics of the patients in the pilot study are shown in Table A1.1.

**Table A1.1** Descriptive statistics of the patient sample in the pilot study.

|  | A (y) | E (y) | EHI SF | BDI FS | VF Ani | VF Let | BNT | WL LE | WL DR | WL IN | WL SA | WL RC | CP CO | CP DR | CP SA | TMT A | TMT B |
| --- | --- | --- | --- | --- | --- | --- | --- | --- | --- | --- | --- | --- | --- | --- | --- | --- | --- |
| *N* | 45 | 45 | 45 | 45 | 45 | 44 | 45 | 45 | 45 | 45 | 45 | 45 | 45 | 45 | 45 | 43 | 36 |
| *M* | 63.71 | 13.66 | 83.61 | 3.47 | -1.11 | -1.01 | -0.10 | -1.30 | -1.04 | -0.53 | -1.35 | -0.92 | -0.76 | -1.09 | -0.64 | -1.07 | -1.21 |
| *SD* | 13.04 | 2.70 | 33.59 | 3.37 | 1.47 | 1.33 | 1.24 | 1.25 | 1.23 | 1.30 | 2.49 | 1.43 | 1.27 | 1.71 | 1.70 | 1.55 | 1.37 |

*Notes.* A = age (in years); E = education (in years); EHI SF = Edinburgh Handedness Inventory - Short Form (Veale, 2014); BDI FS = Beck Depression Inventory - Fast Screen (German version: Kliem & Brähler, 2013). CERAD-Plus Neuropsychological Test Battery (*z* scores; Morris et al., 1988; Aebi, 2002): VF Ani = Animal Fluency (semantic); VF Let = Letter Fluency (phonemic); BNT = Abbreviated Boston Naming Test; WL LE = Word List Learning; WL DR = Word List Delayed Recall; WL IN = Word List Intrusions; WL SA = Word List Savings; WL RC Word List Recognition; CP CO = Construction Practice Copy; CP DR = Construction Practice Delayed Recall; CP SA = Construction Practice Savings; TMT A = Trail Making Test A; TMT B = Trail Making Test B.

First, we analyzed the correlations between the CERAD-Plus scores and the m5PD and mWFC measures of interest (i.e., productivity, accuracy, both operationalized as in the Methods section of the main article, with m5PD accuracy being restricted to design repetitions), and the results are shown in Table A1.2 (Pearson's *r* corrected for age, education, and gender). Inspection reveals that both CERAD-Plus Verbal Fluency scores (semantic, phonemic) correlate with m5PD and mWFC productivity, suggesting some degree of convergent validity. The m5PD productivity also correlated with confrontation naming, constructional praxis, and TMT A scores. The mWFC productivity also correlated with confrontation naming, as well as with learning, delayed recall and recognition on the word list, constructional praxis, and both TMT scores, suggesting convergence with verbal memory, visuoconstructional abilities, and processing speed. In contrast to productivity, neither m5PD nor mWFC accuracy showed consistent patterns of correlation with CERAD-Plus scores, suggesting that these variables capture interindividual variance that is not shared with the bulk of CERAD-Plus scores. Interestingly, however, the only m5PD accuracy correlation emerged with a cross-domain CERAD-Plus score, i.e., letter (phonemic) fluency, and mWFC accuracy also correlated with a cross-domain CERAD-Plus score, i.e., visuoconstructional abilities.

**Table A1.2** Correlations between CERAD-Plus scores and study measures.

|  | | **m5PD  productivity** | | **mWFC  productivity** | | **m5PD  accuracy** | | **mWFC  accuracy** | |
| --- | --- | --- | --- | --- | --- | --- | --- | --- | --- |
| VF Ani | (45) | 0.51 | *** | 0.42 | ** | -0.28 |  | -0.19 |  |
| VF Let | (44) | 0.61 | *** | 0.54 | *** | -0.37 | * | -0.26 |  |
| BNT | (45) | 0.40 | ** | 0.49 | *** | -0.20 |  | -0.28 |  |
| WL LE | (45) | 0.15 |  | 0.40 | ** | 0.09 |  | -0.25 |  |
| WL DR | (45) | 0.08 |  | 0.46 | ** | -0.08 |  | -0.24 |  |
| WL IN | (45) | 0.04 |  | -0.21 |  | 0.08 |  | 0.27 |  |
| WL SA | (45) | -0.05 |  | 0.25 |  | -0.14 |  | -0.24 |  |
| WL RC | (45) | -0.23 |  | 0.34 | * | 0.04 |  | 0.04 |  |
| CP CO | (45) | 0.42 | ** | 0.38 | * | -0.25 |  | -0.37 | * |
| CP DR | (45) | 0.13 |  | 0.43 | ** | -0.12 |  | -0.25 |  |
| CP SA | (45) | -0.05 |  | 0.29 |  | -0.06 |  | -0.13 |  |
| TMT A | (43) | -0.36 | * | -0.45 | ** | 0.03 |  | 0.11 |  |
| TMT B | (36) | -0.26 |  | -0.52 | ** | 0.22 |  | 0.41 | * |

*Notes.*  Pearson's partial correlations conditioned on variables: age, education, gender. m5PD = Modified Five Point Design Task, mWFC = Modified Word Fragment Completion Task; productivity, accuracy. CERAD-Plus raw scores (N in brackets): VF Ani = Animal fluency; VF Let = Letter fluency; BNT = Abbreviated Boston Naming Test; WL LE = Word list learning; WL DR = Word list delayed recall; WL IN = Word list intrusions; WL SA = Word list savings; WL RC Word list recognition; CP CO = Constructional praxis copy; CP DR = Constructional praxis delayed recall; CP SA = Constructional praxis savings; TMT A = Trail Making Test A; TMT B = Trail Making Test B. * *p* < .05, ** *p* < .01, *** *p* < .001

Second, we analyzed the split-half reliabilities and intercorrelations of the m5PD and mWFC measures of interest. The results are presented in Table A1.3. All measures of interest showed reasonably good split-half reliabilities, with coefficients around 0.80. The two productivity measures were positively correlated. On both tasks (m5PD, mWFC), productivity was negatively correlated with accuracy, but none of the between-task correlations between the remaining measures of interest differed from zero.

**Table A1.3** Split-half reliability estimates and correlations among study measures.

|  | **m5PD  productivity** | **mWFC  productivity** | **m5PD  accuracy** | **mWFC  accuracy** |
| --- | --- | --- | --- | --- |
| **m5PD productivity** | 0.88 |  |  |  |
| **mWFC productivity** | 0.44 ** | 0.86 |  |  |
| **m5PD accuracy** | -0.45 ** | -0.14 | 0.79 |  |
| **mWFC accuracy** | -0.28 | -0.54 *** | 0.24 | 0.85 |

| *Notes.*   On diagonal: Split-half reliabilities (*r_SB_*; Steinke & Kopp, 2020). Off diagonal: Pearson's partial correlations conditioned on variables: age, education, gender (*N* = 45). m5PD = Modified Five Point Design Task, mWFC = Modified Word Fragment Completion Task; productivity, accuracy. * *p* < .05, ** *p* < .01, *** *p* < .001 |
| --- |
|  |

In summary, both m5PD and mWFC productivity showed significant correlations with verbal fluency scores, indicating some degree of convergent validity, whereas neither m5PD nor mWFC accuracy correlated consistently with CERAD-Plus scores, except for isolated cross-domain (i.e., verbal-spatial) correlations. In addition, all measures of interest showed comparably good reliability. The two productivity measures correlated positively with each other, but negatively with within-task accuracy. The two accuracy measures showed no significant correlation across tasks, suggesting some degree of divergent validity.

**Appendix 2: The modified five-point design (m5PD) fluency task**

**General Instructions**

The following instructions are guidelines, and strict adherence to them is not necessary. Basic instructions should be communicated. However, in the event of a patient error, it is critical that the instructions are followed precisely.

The examiner sits facing the patient. The patient is given the front sheet of the test booklet. The sheet should be placed in a central position in front of the patient. The order of completion is guided by a "viewing window" (3x3 cm). It should be noted if the patient is using the non-dominant hand.

The time allowed is five minutes (excluding instruction and scoring). The timer should not be stopped once the test has begun. The timer should be kept running even if the patient has additional questions or if there are brief interruptions by the examiner for error correction. Error correction is allowed only once within the first 5 boxes (a more detailed error correction procedure is explained below). If the five minutes run out while the patient is still working on a box, the patient may complete that box. The completed box is then eligible for full scoring in the analysis.

After 3 minutes, the patient is reminded of the remaining time ("You only have two minutes left."). This is repeated after 4 minutes ("You only have one minute left.").

If patients express concern that they can't think of another idea and there is still time, they can be reassured with non-specific encouragement (e.g., "You still have time - maybe you can come up with another idea"). No specific instructions are allowed (e.g., "Try a simple design"). After approximately 10 seconds of inaction, patients are asked to select the next design that immediately comes to mind in order to proceed to the next box. If the patient asks if a pattern is correct, the examiner may only respond with a non-specific instruction (e.g., "You can make a pattern by connecting 2, 3, or 4 points with a line. Each pattern can be drawn only once").

The quality of the drawings is not important for the analysis. Unsteady lines, inaccurate point-to-point connections, etc. are not penalized as long as it is clear that the patient is following the rules. After some experience with the test, the examiner should be able to distinguish poor drawing ability from cognitive difficulties with the task. Qualitative observation is important: Does the patient draw slowly throughout the task? Does the patient produce fewer ideas towards the end of the task? Does the patient explicitly or implicitly indicate that he has run out of ideas?

**Specific Instructions**

**(Keep the order of the instructions and demonstration; use clear and concise language; demonstrate as much as possible)**

**1. Here you see a sheet with many small boxes (show both sides of the sheet). All boxes contain exactly 5 dots - the 5 dots are always arranged in exactly the same way.**

**2. Your task is to draw as many different patterns in the boxes as possible. No two designs should be the same. In the end, you will have created many different designs without repeating any of them.**

**3. You can draw a pattern by connecting 2, 3 or 4 nearest neighbor points inside the box. (Turn to the front page) I will show you a valid example (put a sliding window on top of the upper left box). This is a relatively simple example where only 2 nearest neighbor points are connected, but it is not important whether the designs are simple or complicated. The only important thing is that you come up with a lot of different designs.**

**There are some additional rules I'd like to explain now.**

**4. If you draw an exact copy of a pattern (see upper central example), the repeated pattern cannot be counted and is defined as invalid. If you add another line (draw a line connecting the two top points), the newly generated design is valid again because it has new features. Also, if you connect two points that are different from the points in the upper left example (e.g., connecting the two points on the left side), the design is valid because the line is in a different position.**

**5. A construction is invalid if all 5 points are connected (see upper right example).**

**6. The design must consist of a single line with exactly one start point and one end point. Therefore, this example is invalid (see middle left example).**

**7. Since every design must have exactly one start and one end point, this example (see middle central example) is also invalid with more than 2 start/end points.**

**8. A design is invalid if there is no visible start and end (see middle right example).**

**9. A design is invalid if two points from different boxes are connected (see lower left and central examples). I will guide you from box to box with this "sliding window" (show the "sliding window") to prevent this from happening. On the one hand, the sliding window allows you to concentrate on the correct box. On the other hand, it prevents you from seeing the previously drawn boxes. So be sure to keep them in mind.**

**10. A design is invalid if there is no straight point-to-point connection (e.g. the line is wavy or has corners) (see lower right example).**

**11. You have exactly 5 minutes to complete the task; I will be timing you. You should try to draw as many designs as possible in the 5 minutes - it's okay if you don't reach the end.**

**Do you have any questions? (If there are any questions, repeat the relevant part of the instructions. If patients ask if they are allowed to draw the valid examples shown, the answer is "yes").**

**Go! (Start the timer for 5 minutes without interruption)**

Interventions after the timer has started:

Only if the first error occurs within the first five patterns drawn by the patient (e.g. if the patient draws his first pattern again in the third position), he will be informed about this once, stating the error. The error is not pointed out until the next pattern is started. If the patient then corrects the error, the pattern still counts as an error (do not forget to make a note of this at the end of the test - otherwise it may not be remembered at a later evaluation). Otherwise, all self-corrections are allowed and do not count as errors.

Any further errors - even if they occur within the first five patterns drawn by the patient and/or represent a different type of error - will not be pointed out. If the patient makes a first error after drawing five first correct patterns, it will not be highlighted.

**Figure A2.1** Front page of the m5PD test booklet (showing the instructed examples).

**
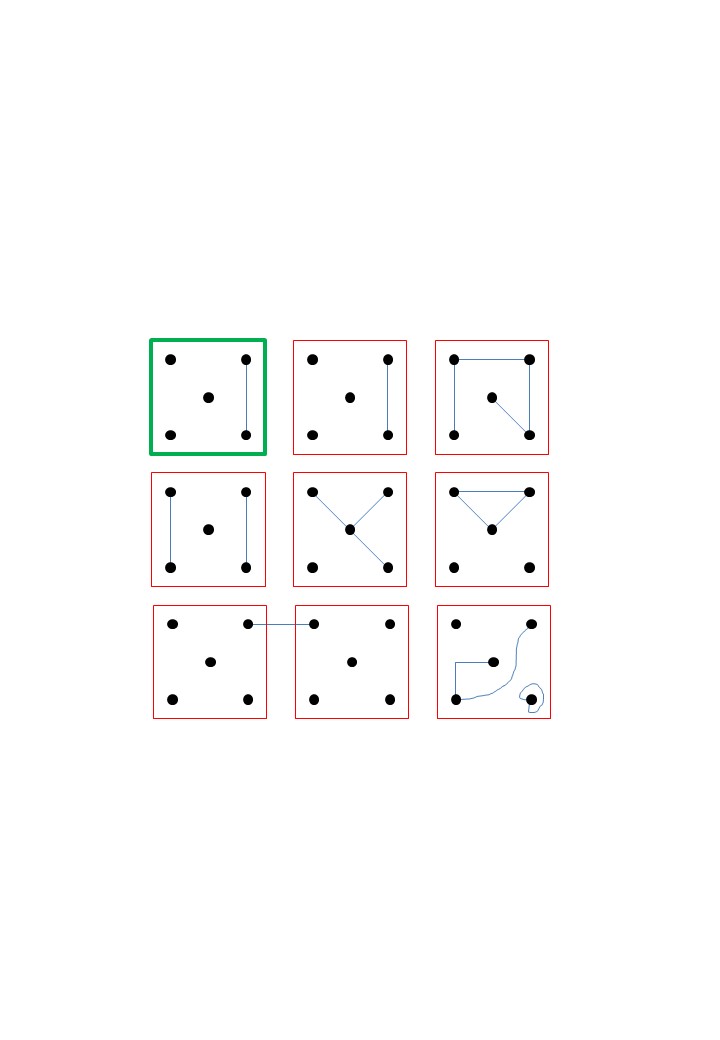
**

**Figure A2.2** All 54 items of the m5PD task (pages 2 and 3 of the test booklet).


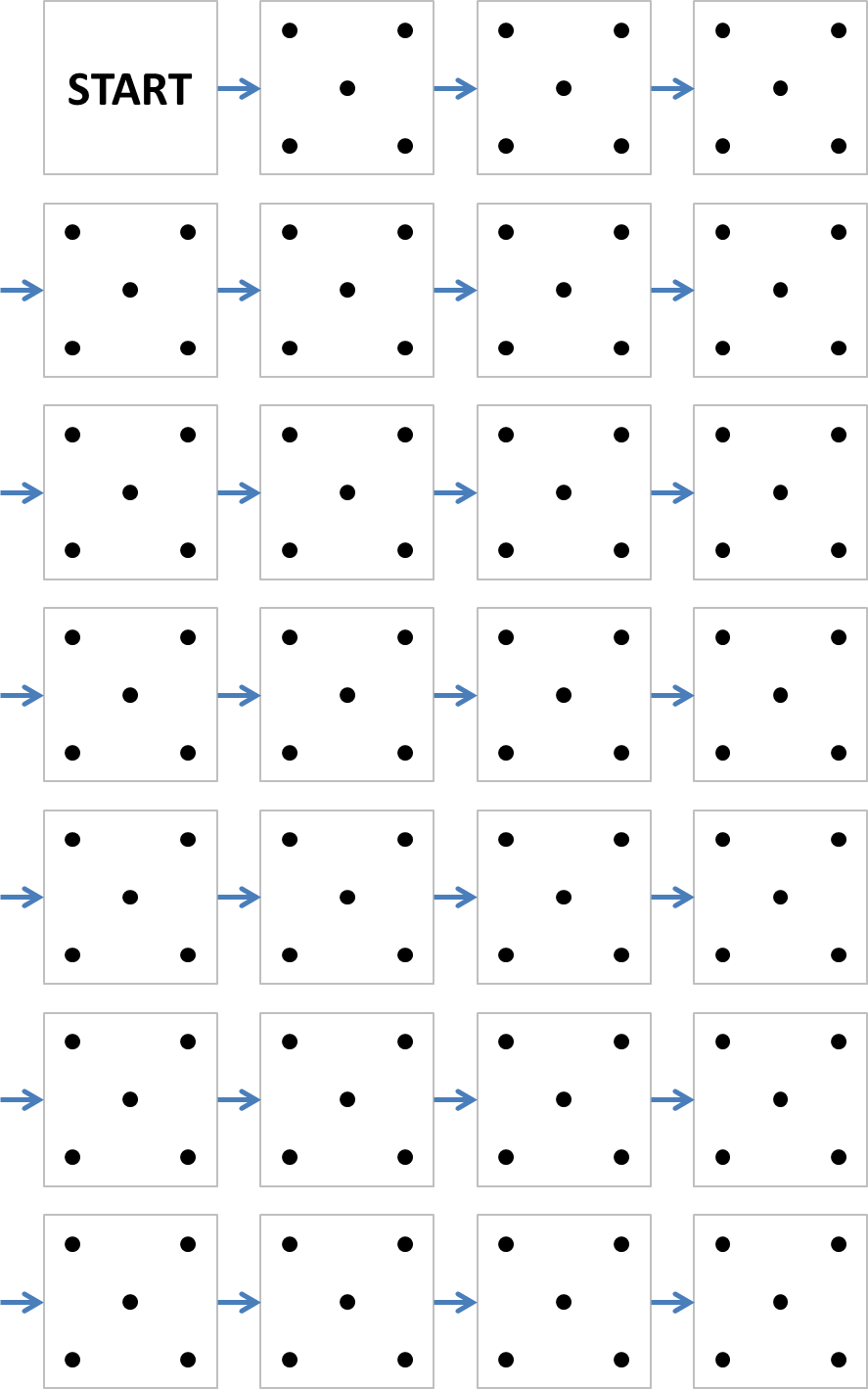

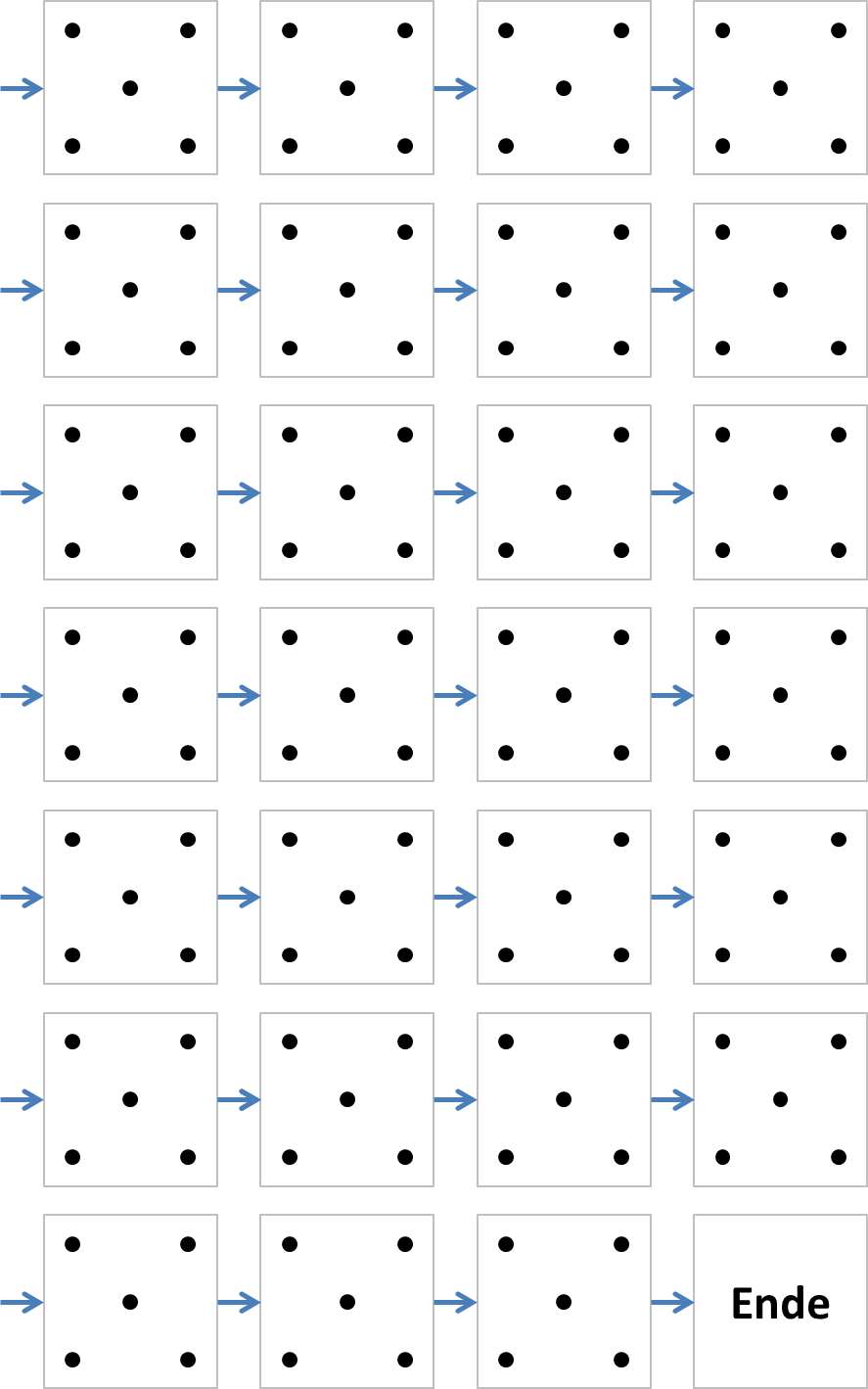


**Figure A2.3** All 54 valid m5PD designs.

| 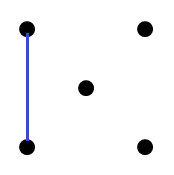 | 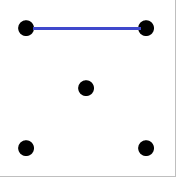 | 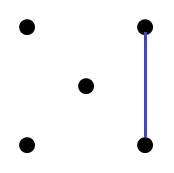 | 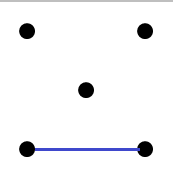 |
| --- | --- | --- | --- |
| 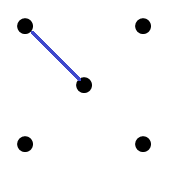 | 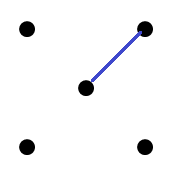 | 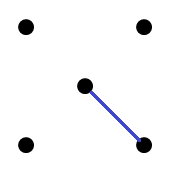 | 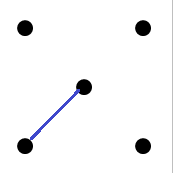 |
| 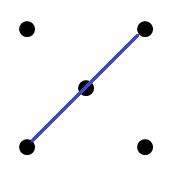 | 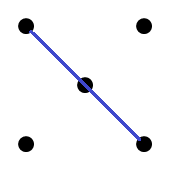 |  |  |
| 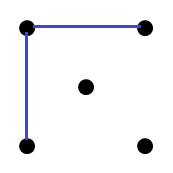 | 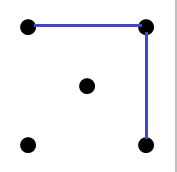 | 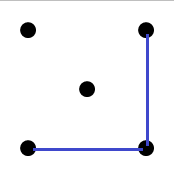 | 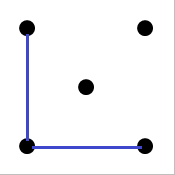 |
| 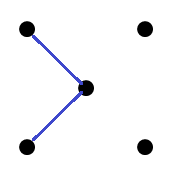 | 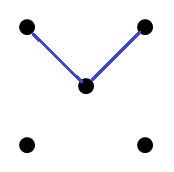 | 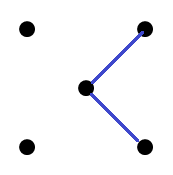 | 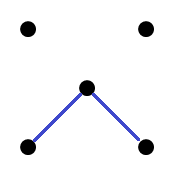 |
| 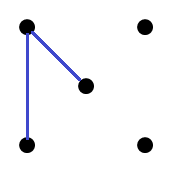 | 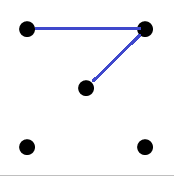 | 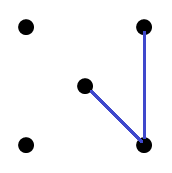 | 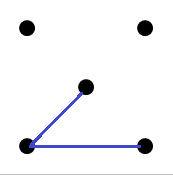 |
| 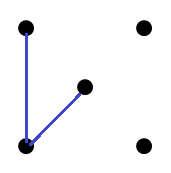 | 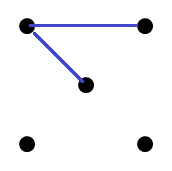 | 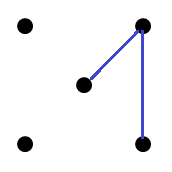 | 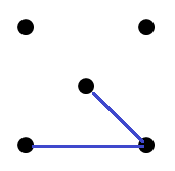 |
| 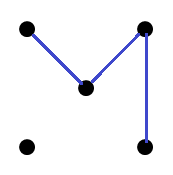 | 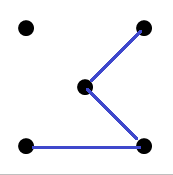 | 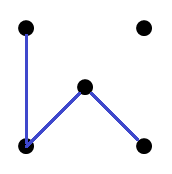 | 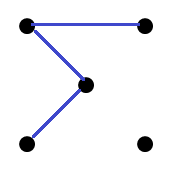 |
| 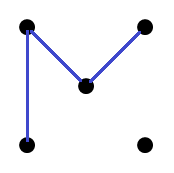 | 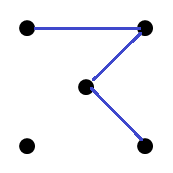 | 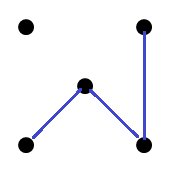 | 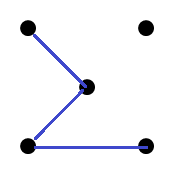 |
| 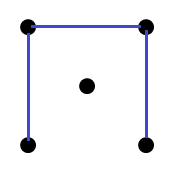 | 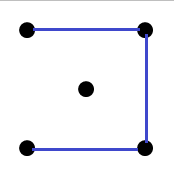 | 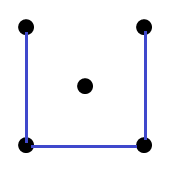 | 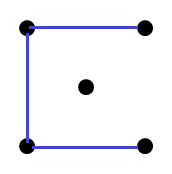 |
| 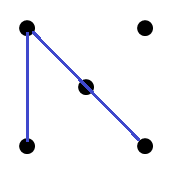 | 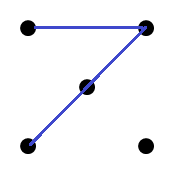 | 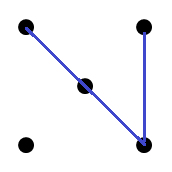 | 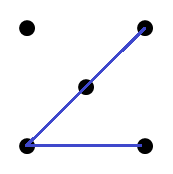 |
| 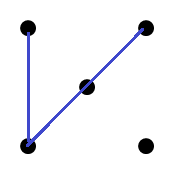 | 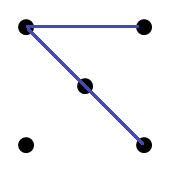 | 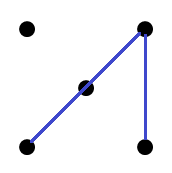 | 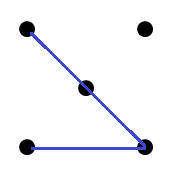 |
| 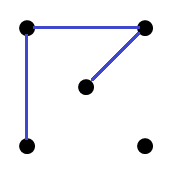 | 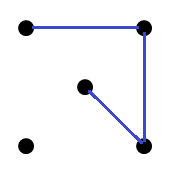 | 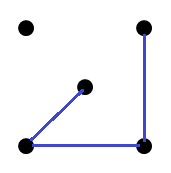 | 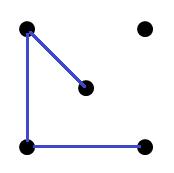 |
| 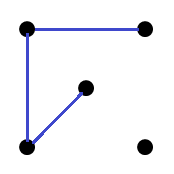 | 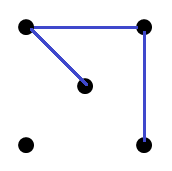 | 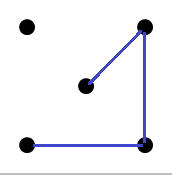 | 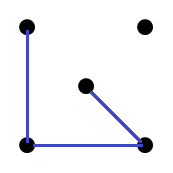 |

**Appendix 3: The modified word-fragment completion (mWFC) task**

**General Instructions**

The following instructions are guidelines, and strict adherence to them is not necessary. Basic instructions should be given. However, in the event of a patient error, it is critical that the instructions be followed precisely.

The examiner sits facing the patient. The patient is given the front sheet of the test booklet. The sheet should be placed in a central position in front of the patient. The order of completion is guided by a "sliding window". The "vowel board" (top left "E", top right "O", center left "Y", center right "A", bottom left "U", bottom right "I") should be placed to the right of the sheet (patient's perspective) if the patient has suffered a right cerebral infarction. This allows the patient to select a vowel with the right hand. The "vowel board" should be placed to the left of the page (patient's perspective) if the patient has suffered a left cerebral infarction. This allows the patient to select a vowel with the left hand.

The time allowed is five minutes (excluding instruction and scoring). The timer should not be stopped once the test has begun. The timer should be kept running even if the patient has additional questions or if there are brief interruptions by the examiner for error correction. Error correction is allowed only once within the first 5 boxes (a more detailed error correction procedure is explained below). If the five minutes run out while the patient is still working on a box, the patient may complete that box. The completed box is then eligible for full scoring in the analysis.

After 3 minutes, the patient is reminded of the remaining time ("You only have two minutes left."). This is repeated after 4 minutes ("You only have one minute left.").

If patients express concern that they can't think of another idea and there is still time left, they can be reassured with non-specific encouragement (e.g., "You still have time - maybe you can come up with another idea"). After about 10 seconds, patients are asked to select the next immediate vowel that comes to mind in order to move on to the next box. In no case are specific cues allowed (e.g., "Try the vowel X."). If the patient asks if the chosen vowel is correct, a nonspecific rendition of that part of the instructions should be given.

**Specific Instructions**

1. **(Keep the order of the instructions and demonstration; use clear and concise language; demonstrate as much as possible)**
2. **You are now looking at a page in front of you. You can see that it consists of several boxes filled with letters. (Show the page only briefly to prevent the patient from solving a box before the timer starts). In each box you will find a consonant at the beginning and end - leaving a blank in the middle.**
3. **Your task is to fill in the blank with a vowel to make a proper noun.**
4. **You even get some help (show the vowel board and read all the vowels). You can only choose one vowel per box - the Y is not a vowel.**
5. **Let's look at a first example together (position the slider on the page with example 1). Do you have any idea what noun could be made with these consonants - "B" and "R"? Which vowel would fit? You can see that you could choose the vowel "A", because it makes the word "BAR". (The examiner should use the vowel board).**
6. **The vowel you choose forms a noun with the two consonants. But if the two consonants are repeated, you cannot make the same word again. You must choose a different vowel to create a different word. Looking at this example - you would not be allowed to choose "BAR" again - an alternative here would be the vowel "O" to make "BOR" - a chemical substance.**
7. **The next example (pointing to "DER ") would be incorrect because it is not a noun but an article.**
8. **This example (pointing to "TYP ") is also incorrect because "Y" is not a vowel.**
9. **Unfortunately, names are not allowed either. (Point to "ROM " and explain that it is the name of the capital of Italy).**
10. **We are not looking for English words either (point to "CAT" and explain that this is the English word for "cat").**
11. **Abbreviations are also not allowed (point to “KAT” and explain that this would be the abbreviation for Katalysator^[[1]](#footnote-1)^)**
12. **You have exactly 5 minutes to complete this task, I'm going to set a timer. I will guide you using the sliding window, but I am only allowed to move to the next box if you have completed the previous one. So, if you get stuck and can't think of a correct vowel, just pick a random one and we can move on to the next box.**
13. **Do you have any questions?**
14. **Go! (Start the timer for 5 minutes without interruption)**

After the timer is set, the examiner may only interrupt the patient under the following circumstances:

Only the first error is pointed out to the patient and only if it occurred within the first five boxes.

No other error should be pointed out to the patient, even if it occurred within the first 5 boxes and/or is a different type of error. If patients make their first error after the first 5 boxes, they should not be made aware of it.

If patients express that they cannot think of another vowel, the examiner should use non-specific encouragement (e.g., "Try again, there's still time, maybe you can think of something else", etc.). Under no circumstances should the examiner give specific hints (e.g., "Try the vowel A").

**Figure A3.1** Front page of the mWFC test booklet (showing the instructed examples).


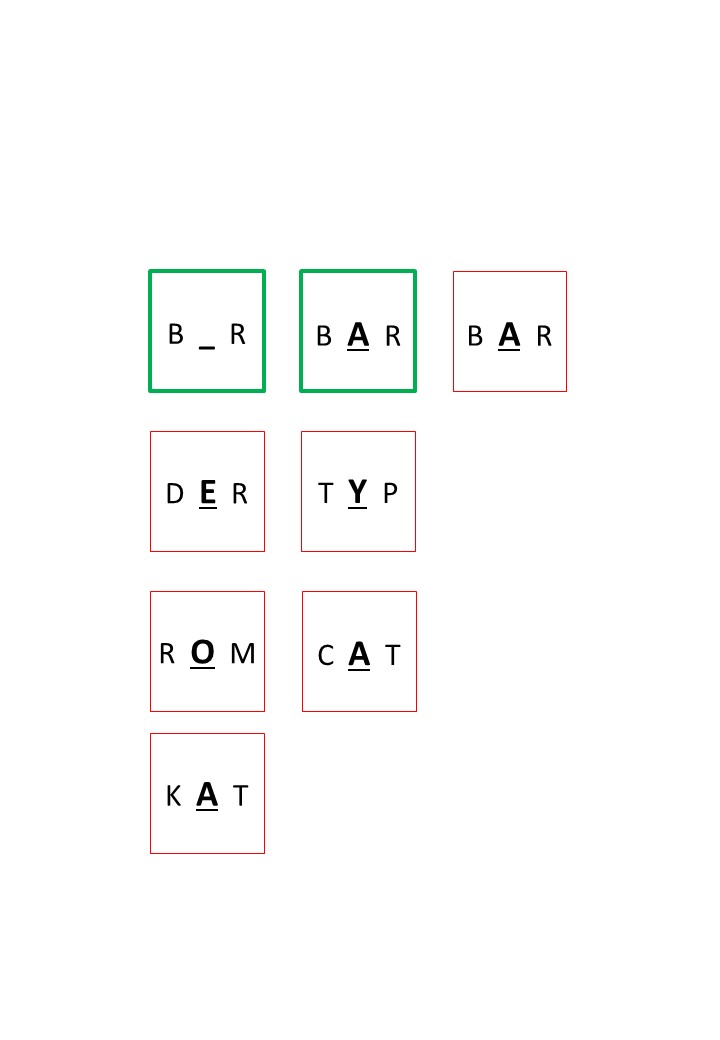


**Figure A3.2** All 54 items of the mWFC task (pages 2 and 3 of the test booklet).


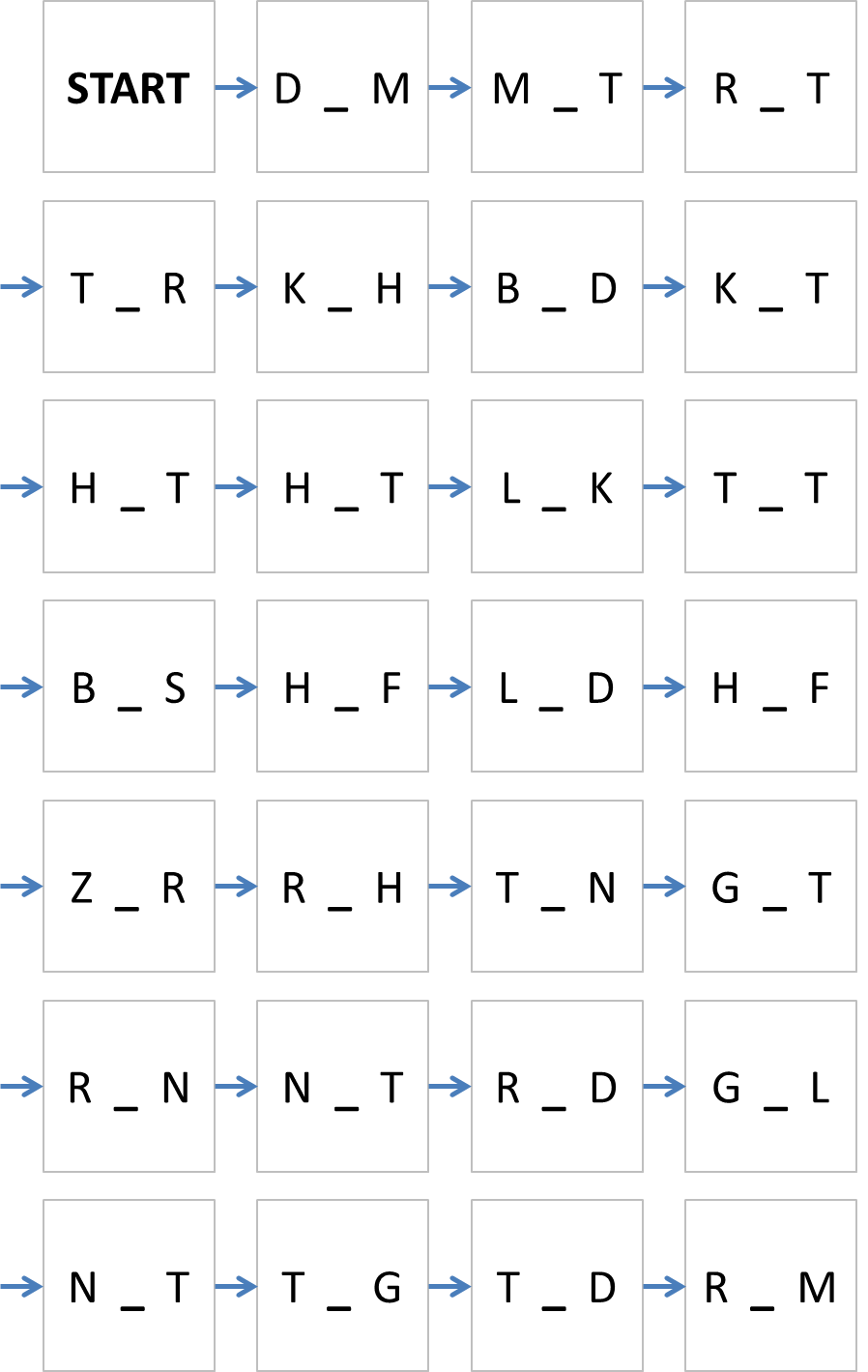

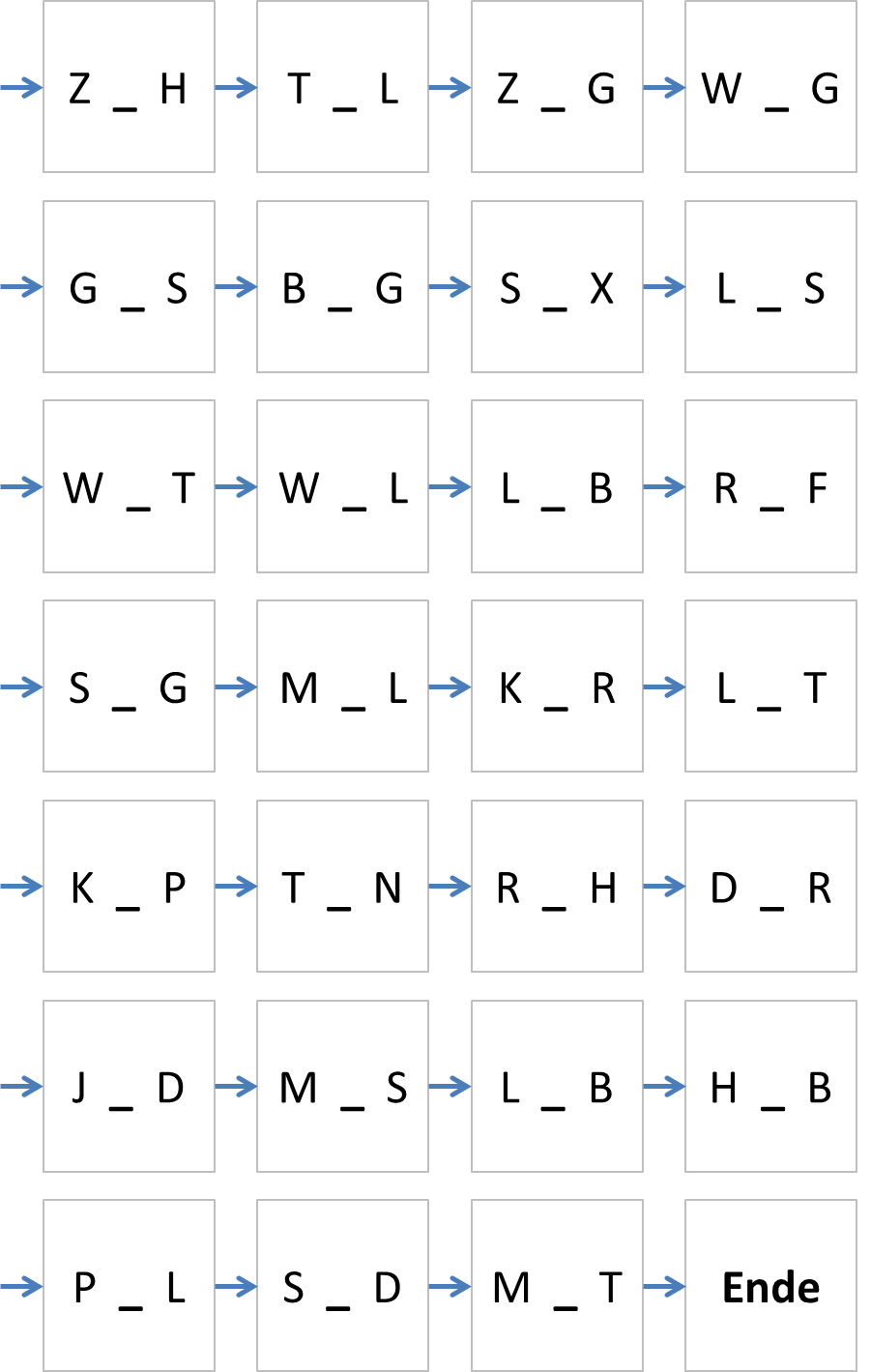


**Table A3.1** Correct nouns (in bold, plus their English translations) and possible distractors on all 54 items of the mWFC task, with log-scaled word frequencies.

| **No** | **Item** | **A** | **E** | **I** | **O** | **U** | **Translation** |  |  |  |  |  |  |  |  |  |  |
| --- | --- | --- | --- | --- | --- | --- | --- | --- | --- | --- | --- | --- | --- | --- | --- | --- | --- |
| **1** | D _ M | DAM | DEM 7 | DIM | **DOM 4** | DUM | cathedral |  |  |  |  |  |  |  |  |  |  |
| **2** | M _ T | MAT | **MET 3** | MIT 7 | MOT | **MUT 4** | mead/courage |  |  |  |  |  |  |  |  |  |  |
| **3** | R _ T | **RAT 5** | RET | RIT | ROT 5 | RUT | advice |  |  |  |  |  |  |  |  |  |  |
| **4** | T _ R | TAR | TER | TIR | **TOR 5** | TUR | gate |  |  |  |  |  |  |  |  |  |  |
| **5** | K _ H | KAH | KEH | KIH | KOH | **KUH 4** | cow |  |  |  |  |  |  |  |  |  |  |
| **6** | B _ D | **BAD 5** | BED | BID | BOD | BUD | bath |  |  |  |  |  |  |  |  |  |  |
| **7** | K _ T | KAT 4 | KET | KIT 3 | **KOT 3** | KUT | dung |  |  |  |  |  |  |  |  |  |  |
| **8** | H _ T | HAT 7 | HET | **HIT 4** | HOT 3 | **HUT 4** | hit/hat |  |  |  |  |  |  |  |  |  |  |
| **9** | H _ T | HAT 7 | HET | **HIT 4** | HOT 3 | **HUT 4** | hit/hat |  |  |  |  |  |  |  |  |  |  |
| **10** | L _ K | LAK | LEK | LIK | **LOK 4** | LUK 2 | locomotive |  |  |  |  |  |  |  |  |  |  |
| **11** | T _ T | **TAT 5** | TET | TIT | TOT 5 | TUT 6 | act |  |  |  |  |  |  |  |  |  |  |
| **12** | B _ S | BAS | BES | BIS 6 | BOS | **BUS 5** | bus |  |  |  |  |  |  |  |  |  |  |
| **13** | H _ F | HAF | HEF | HIF | **HOF 5** | **HUF 3** | yard/hoof |  |  |  |  |  |  |  |  |  |  |
| **14** | L _ D | LAD | LED | **LID 3** | LOD | LUD | lid |  |  |  |  |  |  |  |  |  |  |
| **15** | H _ F | HAF | HEF | HIF | **HOF 5** | **HUF 3** | yard/hoof |  |  |  |  |  |  |  |  |  |  |
| **16** | Z _ R | **ZAR 3** | ZER | ZIR | ZOR | ZUR 6 | tsar |  |  |  |  |  |  |  |  |  |  |
| **17** | R _ H | **RAH 2** | **REH 4** | RIH | ROH 4 | RUH 5 | yardarm/deer |  |  |  |  |  |  |  |  |  |  |
| **18** | T _ N | TAN 4 | TEN | TIN | **TON 5** | **TUN 4** | tone/action |  |  |  |  |  |  |  |  |  |  |
| **19** | G _ T | GAT | GET | GIT | GOT | **GUT 6** | property |  |  |  |  |  |  |  |  |  |  |
| **20** | R _ N | RAN 4 | **REN 3** | RIN 3 | RON | RUN 4 | reindeer |  |  |  |  |  |  |  |  |  |  |
| **21** | N _ T | NAT | NET | NIT | **NOT**  **5** | **NUT**  **3** | poverty/groove |  |  |  |  |  |  |  |  |  |  |
| **22** | R _ D | **RAD**  **5** | RED | RID | ROD | RUD | wheel |  |  |  |  |  |  |  |  |  |  |
| **23** | G _ L | GAL | **GEL**  **3** | GIL | GOL | GUL | gel |  |  |  |  |  |  |  |  |  |  |
| **24** | N _ T | NAT | NET | NIT | **NOT**  **5** | **NUT**  **3** | poverty/groove |  |  |  |  |  |  |  |  |  |  |
| **25** | T _ G | **TAG**  **6** | TEG | TIG | TOG | TUG | day |  |  |  |  |  |  |  |  |  |  |
| **26** | T _ D | TAD | TED | TID | **TOD**  **5** | TUD | death |  |  |  |  |  |  |  |  |  |  |
| **27** | R _ M | RAM 4 | REM 3 | RIM | ROM 5 | **RUM**  **3** | rum |  |  |  |  |  |  |  |  |  |  |

| **No** | **Item** | **A** | **E** | **I** | **O** | **U** | **Translation** |
| --- | --- | --- | --- | --- | --- | --- | --- |
| **28** | Z _ H | ZAH | **ZEH 3** | ZIH | ZOH | ZUH | toe |
| **29** | T _ L | **TAL 4** | TEL 6 | TIL | TOL | TUL | valley |
| **30** | Z _ G | ZAG | ZEG | ZIG 3 | ZOG 5 | **ZUG 5** | train |
| **31** | W _ G | WAG | **WEG 5** | WIG | WOG | WUG | path |
| **32** | G _ S | **GAS 4** | GES | GIS | GOS | GUS | gas |
| **33** | B _ G | BAG | BEG | BIG | BOG 4 | **BUG 4** | bow |
| **34** | S _ X | SAX | **SEX 4** | SIX | SOX | SUX | sex |
| **35** | L _ S | LAS | LES | LIS | **LOS 5** | LUS | lot |
| **36** | W _ T | WAT | WET | WIT | WOT | **WUT 4** | anger |
| **37** | W _ L | **WAL 4** | WEL | WIL | WOL | WUL | whale |
| **38** | L _ B | **LAB 1** | LEB | LIB | **LOB 4** | LUB | rennet/praise |
| **39** | R _ F | RAF (ne) | REF | RIF | ROF | **RUF 5** | call |
| **40** | S _ G | SAG | SEG | SIG | **SOG 3** | SUG | suck |
| **41** | M _ L | **MAL 5** | MEL | MIL | MOL 3 | MUL | mark |
| **42** | K _ R | KAR | KER | KIR | KOR | **KUR 4** | cure |
| **43** | L _ T | LAT | LET | LIT | **LOT 3** | LUT | bob |
| **44** | K _ P | **KAP 3** | KEP | KIP | KOP | KUP | cape |
| **45** | T _ N | TAN  4 | TEN | TIN | **TON 5** | **TUN 4** | tone/action |
| **46** | R _ H | **RAH 2** | **REH 4** | RIH | ROH 4 | RUH 5 | yardarm/deer |
| **47** | D _ R | DAR | DER 7 | DIR 6 | DOR | **DUR 3** | major (music) |
| **48** | J _ D | JAD | JED | JID | **JOD 3** | JUD | iodine |
| **49** | M _ S | MAS | MES | MIS | MOS | **MUS 3** | purée |
| **50** | L _ B | **LAB 1** | LEB | LIB | **LOB 4** | LUB | rennet/praise |
| **51** | H _ B | HAB 4 | HEB | HIB | HOB  5 | **HUB 3** | throw |
| **52** | P _ L | PAL 1 | PEL | PIL | **POL 3** | PUL | pole |
| **53** | S _ D | SAD | SED  4 | SID | SOD | **SUD 3** | brew |
| **54** | M _ T | MAT | **MET 3** | MIT  7 | MOT | **MUT 4** | mead/courage |

*Notes.* Valid CVC nouns appear in bold; ambiguous CVC trigrams are highlighted with a gray background; non-nouns appear in light green; proper names and acronyms appear in dark green. Noun frequency on a 7-point logarithmic scale (1(rare)-7(frequent); source: DWDS – Digitales Wörterbuch der deutschen Sprache. Das Wortauskunftssystem zur deutschen Sprache in Geschichte und Gegenwart, hrsg. v. d. Berlin-Brandenburgischen Akademie der Wissenschaften, <https://www.dwds.de/>, abgerufen am 02.06.2023. (ne) = non-existent in the DWDS data base, but a well-known acronym (e.g., ‘RAF’).

**Appendix4: Imaging acquisition variables**

**Table A4.1** Imaging acquisition variables for each scanner model.

| Scanner model | SIEMENS Verio | SIEMENS Skyra | SIEMENS Avanto | SIEMENS Aera |
| --- | --- | --- | --- | --- |
| Study id | 4652004 | 4676107 | 4672165 | 4651456 |
| Series number | 5 | 7 | 4 | 7 |
| Repetition time (ms) | 8300 | 6900 | 3900 | 7100 |
| Echo time (ms) | 94 | 98 | 94 | 91 |
| Flip angle | 90 | 90 | 90 | 90 |
| Number of averages | 1 | 2 | 4 | 2 |
| Slice thickness (mm) | 5.5 | 5.5 | 5.5 | 5,5 |
| Slice spacing (mm) | 6.05 | 6.05 | 6.05 | 6.05 |
| Image columns | 162 | 384 | 192 | 384 |
| Image rows | 162 | 384 | 192 | 384 |
| Phase encoding direction | COL | COL | COL | COL |
| Voxel size x (mm) | 1.41975 | 0.625 | 1.25 | 0.625 |
| Voxel size y (mm) | 1.41975 | 0.625 | 1.25 | 0.625 |
| Number of slices | 24 | 25 | 24 | 24 |
| Number of files | 24 | 25 | 24 | 24 |
| Orientation | Axial | Axial | Axial | Axial |

**Appendix5: Supplementary analysis**

We conducted supplementary lesion-symptom mapping analyses while covarying for age, sex, and years of education (see Figure A4.1).

**Figure A5.1:** Lesion clusters after controlling for demographic covariates. The figure shows lesion clusters obtained from SCCAN including age, sex, and years of education that were significantly associated with m5PD (a) and mWFC (b1-3) accuracy. (a) The MNI coordinates of the cluster peak on the m5PD task were: -30,-25.2,27.1. (b1-3) The MNI coordinates of the cluster peaks on the mWFC task were: (b1) 30.4,-9.7,27.8; (b2) 32.6,7.2,27.8; (b3) 37.1,11.7,10.1.


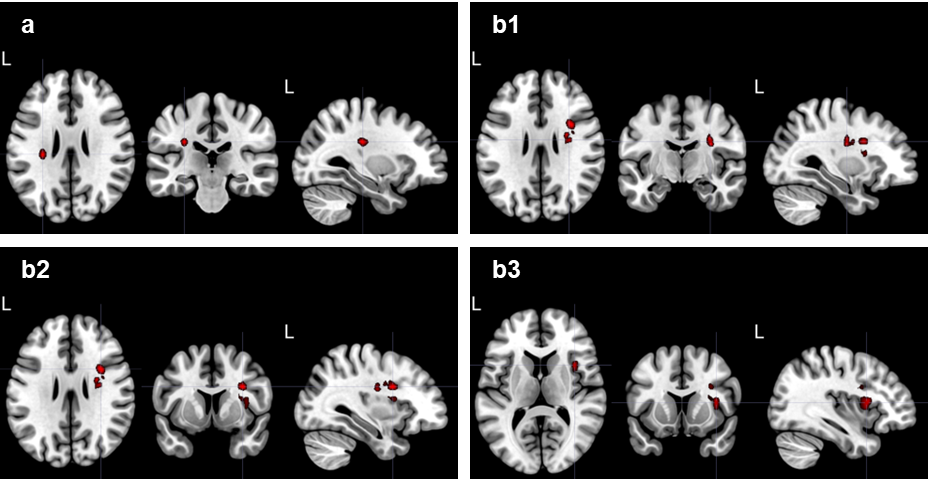


For the spatial fluency (m5PD) task (see Figure A4.1a), incorporating these demographic covariates refined our results. The previously identified right inferior frontal cluster associated with accuracy no longer reached significance. However, the left frontal white matter cluster remained robustly associated with m5PD accuracy, underscoring its critical role in task performance even after accounting for demographics. For the verbal completion (mWFC) task (see Figure A4.1b1-3), the findings were consistent with our original analysis. The right frontal clusters associated with mWFC accuracy persisted even after adjusting for age, sex, and education.

These supplementary analyses demonstrate the stability of core findings while providing a more nuanced understanding of the relationships between lesion location, cognitive outcomes, and demographic influences. By isolating the contribution of lesion location alongside these covariates, we hope to further strengthen the comprehensiveness of the study and its contribution to the literature.

References

1. Veale, J. F. (2014). Edinburgh handedness inventory - short form: a revised version based on confirmatory factor analysis. *Laterality*, *19*, 164-177.
2. Kliem, S. & Brähler, E. (2013). Beck Depressions-Inventar - Fast Screen. Frankfurt/M., Germany: Pearson.
3. Morris, J. C., Mohs, R. C., Rogers, H., Fillenbaum, G., & Heyman, A. (1988). Consortium to establish a registry for Alzheimer's disease (CERAD) clinical and neuropsychological assessment of Alzheimer's disease. *Psychopharmacology Bulletin*, *24,* 641-652.
4. Aebi, C. (2002). *Validierung der neuropsychologischen Testbatterie CERAD-NP: eine Multi-Center Studie* (Doctoral dissertation, University of Basel, Switzerland).
5. Steinke, A., & Kopp, B. (2020). RELEX: An Excel-based software tool for sampling split-half reliability coefficients. *Methods in Psychology*, *2*, 100023.

1. Katalysator = catalyst [↑](#footnote-ref-1)
